# Imaging features of lacrimal gland disease

**DOI:** 10.3389/fopht.2025.1724513

**Published:** 2026-01-07

**Authors:** Carmelo Caltabiano, Khizar Rana, Alexander Buckby, Sandy Patel, Dinesh Selva

**Affiliations:** 1 Discipline of Ophthalmology and Visual Sciences, University of Adelaide, Adelaide, SA, Australia; 2 Department of Ophthalmology, Queen Elizabeth Hospital, Adelaide, SA, Australia; 3 College of Medicine and Public Health, Flinders University, Adelaide, SA, Australia

**Keywords:** adenoid cystic carcinoma, dacryoadenitis, lacrimal gland mass, lacrimal gland tumour, lacrimal gland pathology, pleomorphic adenoma

## Abstract

Lacrimal gland masses represent a diverse group of pathological processes, including inflammatory, lymphoproliferative, and neoplastic lesions. They often present as a palpable mass in the superolateral orbit. There is significant overlap in clinical presentation, and imaging with ultrasound, computed tomography, and magnetic resonance imaging is essential for further characterisation. Key radiological features such as laterality, lobe involvement, lesion composition, margin definition, enhancement pattern, and associated bony changes can significantly narrow the differential diagnosis. This review will describe the radiological features of lacrimal gland masses to guide decision-making.

## 1 Introduction

Lacrimal gland pathologies pose a diagnostic challenge, often presenting as a palpable mass in the superolateral orbit (1, 2). These lesions encompass a broad spectrum of conditions, including epithelial and mesenchymal neoplasms, inflammatory and autoimmune conditions, lymphoproliferative processes, metastases, and structural abnormalities. Among these, inflammatory and lymphoproliferative disorders are the most common, often bilateral with diffuse involvement of both lobes. In contrast, epithelial neoplasms, benign or malignant, are characteristically unilateral and have a predilection to affect the orbital lobe (2, 3). Pleomorphic adenoma represents the most common benign epithelial tumour, while adenoid cystic carcinoma is the most common malignant type (1).

Radiological imaging is essential for evaluating suspected lacrimal gland pathology. Magnetic resonance imaging (MRI) is the preferred investigation due to its superior soft resolution and higher concordance with histopathology than computed tomography (CT) (42% vs. 12%) (4). In addition, the apparent diffusion coefficient (ADC) obtained from diffusion-weighted imaging (DWI) is a safe, reliable, and non-invasive parameter that can effectively differentiate malignant tumours from benign lacrimal lesions with high sensitivity and specificity, as well as distinguish lymphomas from non-lymphoproliferative masses (4). Fat-suppressed, contrast-enhanced MRI is especially useful for neoplastic and inflammatory conditions due to the gland’s vascularity and characteristic pathological enhancement patterns (5, 6). In contrast, CT remains complementary, primarily for assessing osseous involvement. Ultrasound (US) has limited utility and is not routinely employed.

Osseous changes may be classified in two main categories: expansile remodelling or extensive bone destruction. Slowly expanding lesions, whether benign or malignant, may produce expansile bony remodelling, in which new bone forms after initial destruction, without periosteal disruption (7, 8). In such cases, the tumour mass typically remains cohesive and separated from the bone by fibrosis (7). Bone erosion refers to a focal area of cortical loss or thinning, while scalloping describes bending of the bone (7). Sclerotic change denotes an abnormal increase in bone density adjacent to the tumour (7). In the lacrimal fossa, expansile remodelling may appear as sharply defined cortical indentations or generalised enlargement with cortical thinning (8). In contrast, extensive bone destruction reflects a more aggressive process (7, 8). It results from rapid tumour growth and invasion, during which the connective tissue barrier is breached, and tumour infiltrates the bone marrow (7). This produces lytic change, characterised by reduced bone density (7). Full thickness bone extension may be seen (7).

Imaging features are important in narrowing the differential diagnosis of lacrimal gland lesions and guiding appropriate management. Key radiological features include lesion composition (homogeneous vs heterogeneous, solid vs cystic), lobe involvement (orbital vs palpebral), laterality (unilateral vs bilateral), margin characteristics (well-circumscribed vs infiltrative), gland size (enlargement vs atrophy), the presence of fat, fluid, and calcification, associated bony changes (remodelling vs destruction), posterior margin of the gland (wedge vs rounded), and enhancement patterns (1, 2, 6, 9–11). This review provides an overview of the radiological features of various lacrimal gland masses, aiming to support accurate preoperative diagnosis and inform clinical decision-making.

## 2 Normal lacrimal gland

**Figure 1:**
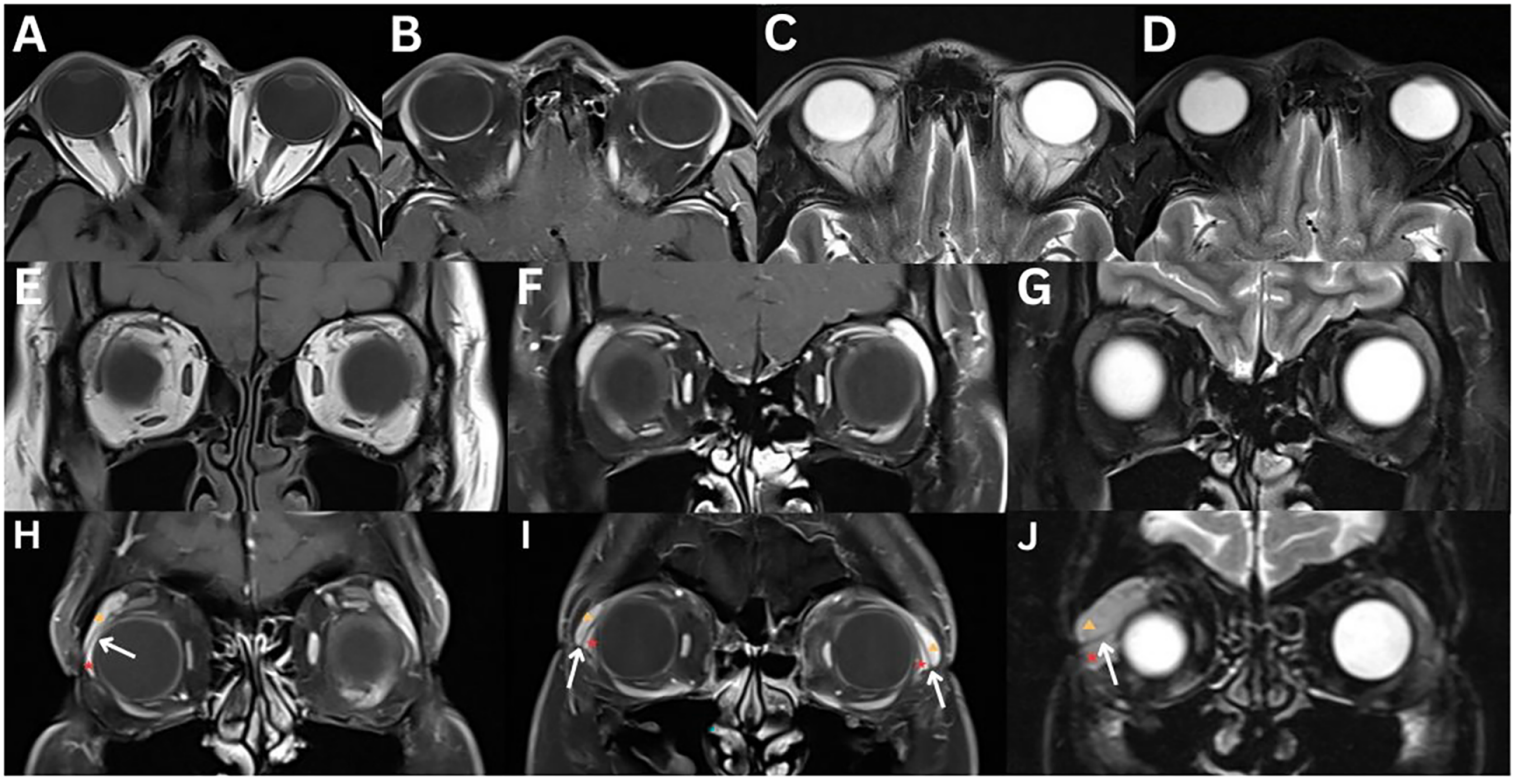
Normal MRI of the lacrimal glands **(A–G)**. T1-weighted axial **(A)** and coronal **(E)**, T1-weighted fat-suppressed contrast-enhanced axial **(B)** and coronal **(F)**, T2-weighted axial **(C)**, and T2-weighted fat-suppressed axial **(D)** and coronal **(G)** images demonstrate a lacrimal gland that is homogeneous in appearance, isointense to the surrounding extraocular muscle, in both T1- and T2-weighted images, and enhances bilaterally. Varying aetiologies of the lacrimal gland **(H–J)**. T1-weighted fat-suppressed contrast-enhanced coronal **(H, I)** and T2-weighted fat-suppressed coronal **(J)** sequences demonstrate the lateral horn of the aponeurosis of the levator palpebrae superioris muscle (white arrow), which separates the lacrimal gland into the orbital lobe (yellow triangle) and the palpebral lobe (red star).

The lacrimal gland is an almond-shaped, eccrine secretory gland responsible for tear production (2). Located in the superolateral aspect of the orbit within the lacrimal fossa of the frontal bone, it lies in the extraconal space adjacent to the superior and lateral rectus muscles. The gland comprises two lobes: the larger orbital lobe posterosuperiorly and the smaller palpebral lobe anteroinferiorly, separated anatomically by the lateral horn of the aponeurosis of the levator palpebrae superioris muscle, which is best visualised on the coronal scans (**Figure 1**) (1, 2, 9, 12, 13). Histologically and developmentally, the lacrimal gland resembles a minor salivary gland, containing both epithelial and lymphoid tissue (6, 9). Gland size is symmetrical (2, 6).

On CT, the lacrimal gland appears isodense to adjacent extraocular muscles and demonstrates symmetric enhancement after contrast administration (1, 2, 6). The gland is located between the orbital fat medially and the orbital bone laterally (2).

On MRI, the gland exhibits intermediate signal on T1- and T2-weighted images, typically appearing homogeneous, although occasionally mildly heterogeneous. Due to its inherent vascularity, the lacrimal gland enhances symmetrically following gadolinium administration (1, 2, 6, 10, 13). On T1-weighted imaging, the orbital septum, which separates the preseptal space from the orbit, appears as a low-signal intensity structure (6). Using DWI sequences, normal gland ADC was 891 ± 103 × 10^–6^ mm^2^/sec in one study (13).

On US, the gland appears hypoechoic with a homogeneous internal echo pattern (14, 15). Its morphology can vary, presenting as oval, circular, diamond-shaped with blunt angles, or irregular in outline (14). It is located anterior to the distal portion of the lacrimal artery and is often barely distinguishable from surrounding orbital fat (15).

## 3 Epithelial tumours

### 3.1 Benign

#### 3.1.1 Pleomorphic adenoma

Pleomorphic adenomas are benign epithelial tumours of the lacrimal gland, accounting for 12-25% of all lacrimal tumours (2, 9, 12). Known as benign mixed tumours, diagnosis requires histopathologic confirmation of both mesenchymal and epithelial components (1). Whilst definitive diagnosis is histological, radiological features are highly characteristic; approximately 90% of pleomorphic adenomas are diagnosed based on clinical and radiological findings alone (11). Nevertheless, they can be indistinguishable from other rare benign lacrimal tumours on imaging, highlighting the importance of biopsy for pathological evaluation (1, 16). Although benign, pleomorphic adenomas may undergo malignant transformation in up to 20% of cases, especially following incomplete excision (6, 11).

**Figure 2:**
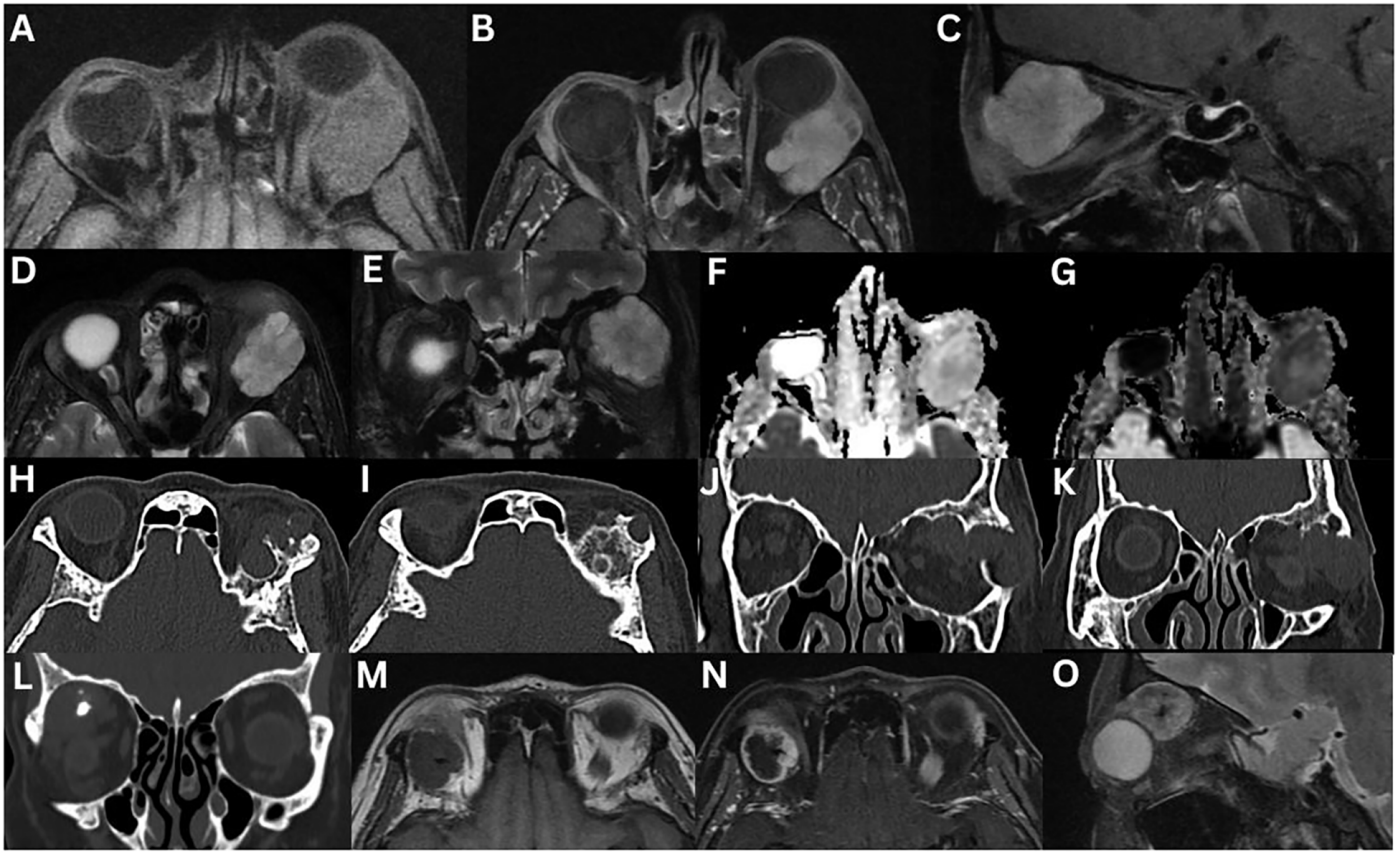
Pleomorphic adenoma of the left lacrimal gland **(A–G)**. T1-weighted axial FLAIR fat-suppressed **(A)**, T1-weighted fat-suppressed contrast-enhanced axial **(B)** and sagittal **(C)**, T2-weighted axial **(D)**, T2-weighted fat-suppress contrast-enhanced coronal **(E)** sequences are demonstrated. A large, irregular, lobulated mass occupies the lacrimal gland. The lesion appears heterogeneous with intermediate signal intensity and shows marked enhancement after contrast administration. There is significant compression and displacement of the lateral rectus muscle in the extraconal space, partially extending intraconally. There is no restricted diffusion on ADC **(F)** and eADC **(G)** sequences. In an 80-year-old female with a pleomorphic adenoma, CT axial **(H, I)** and coronal **(J, K)** views demonstrate the left lacrimal gland mass with bone formation and destruction. In a 55-year-old female with pleomorphic adenoma, coronal CT **(L)** demonstrates intralesional calcifications with no evidence of bony changes. T1-weighted axial **(M)**, T1-weighted fat-suppressed contrast-enhanced axial **(N)**, and T2-weighted sagittal fat-suppressed **(O)** sequences demonstrate an irregularly bordered, heterogeneous mass with intralesional hypointensity with could reflect cystic degeneration, mesenchymal stroma, necrosis, haemorrhage, or mucous/serous fluid collections. There is moderate enhancement following contrast administration. The mass demonstrates a low signal intensity on T1-weighted imaging, and an intermediate signal intensity on T2-weighted imaging.

On CT, pleomorphic adenomas typically appear as unilateral, well-circumscribed, round or oval masses with smooth, regular margins, most often involving the orbital lobe (1, 3, 6, 9, 16–20). Irregular or lobulated contours may rarely occur. Up to 10% of cases are confined to the palpebral lobe (1–3). The tumours demonstrate moderate to marked contrast enhancement (2, 3, 6, 9, 19), and may contain intratumoral calcification (1, 2, 6, 16, 17), or rarely haemorrhage (6). Signal heterogeneity depends on composition and cellularity (1, 10); highly cellular tumours are often homogeneous, while abundant mesenchymal stroma, necrosis, cystic degeneration, or mucous/serous fluid collections cause heterogeneity (10). Cystic degeneration is more common in large, long-standing tumours, presenting as radiolucent regions reflecting myxomatous and/or cartilaginous components, similar to findings in parotid gland pleomorphic adenomas (19–21). Their slow-growing nature can lead to bone scalloping or pressure-related expansion of the lacrimal fossa, without true bone destruction (1–3, 6, 16–20), although advanced cases may show destructive changes (19). Bone formation is rare (**Figure 2**) (22). Enlarging tumours often displace the globe inferonasally (3, 9). In a study of 45 patients with pleomorphic adenomas, CT findings included a well-circumscribed mass (100%), isodense signal relative to surrounding muscle (100%), homogeneity (78%), calcification (13%), globe displacement (73%), bone remodelling (67%), orbital roof defects (11%), bony invasion (2.2%), and no bony change (20%) (23).

**Figure 3:**
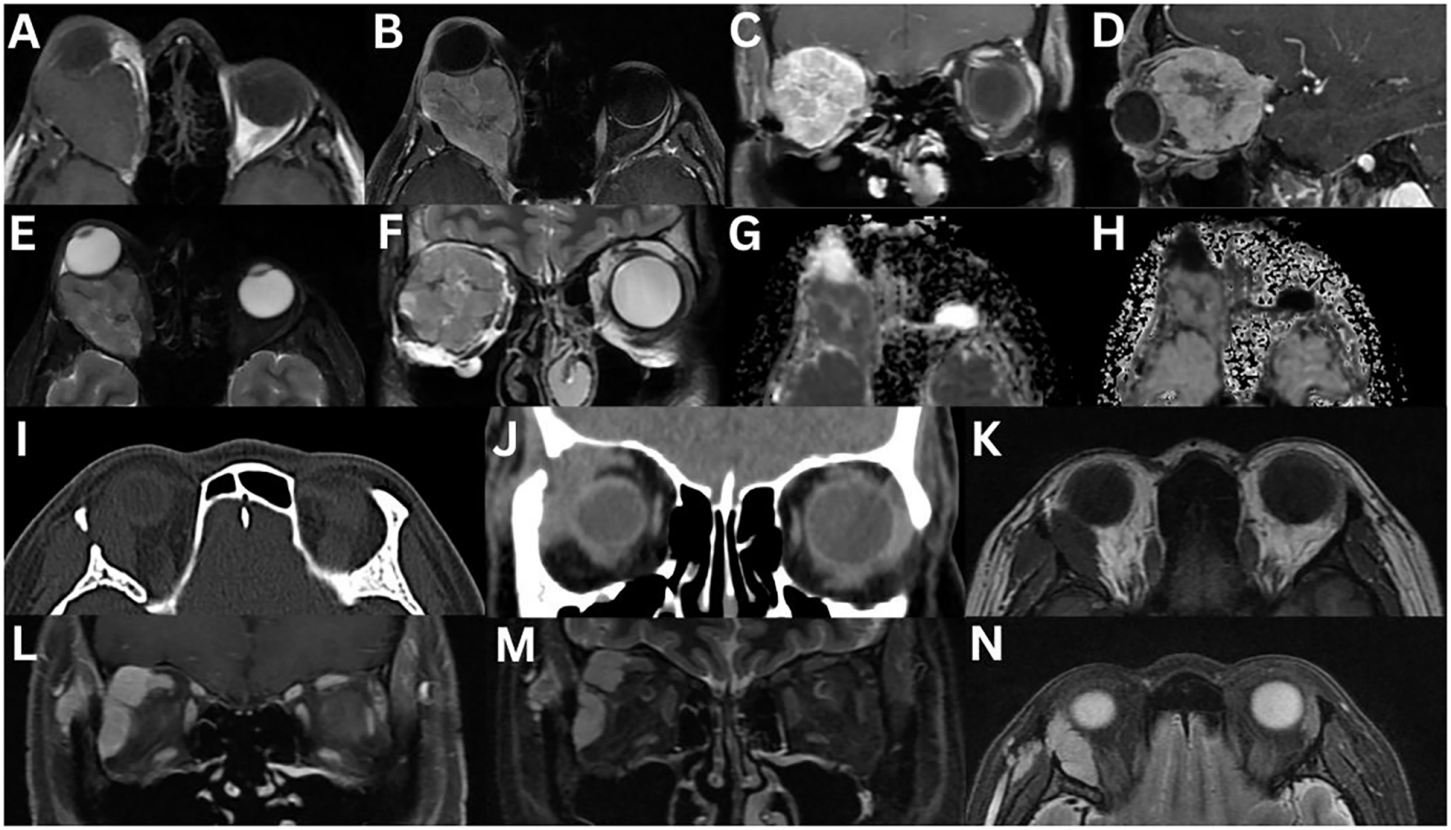
Adenoid cystic carcinoma of the right lacrimal gland **(A–H)**. MRI T1-weighted axial **(A)**, and fat-suppressed contrast-enhanced axial **(B)**, coronal **(C)**, and sagittal **(D)** sequences demonstrate an irregular, lobulated mass with evidence of central necrosis, infiltrating the extraocular muscles and the optic nerve sheath. The lesion is isointense to extraocular muscles on T1-weighted imaging and demonstrates a positive lateral wedge sign. T2-weighted axial **(E)** and coronal **(F)** sequences demonstrate a mass that is hyperintense to extraocular muscles on T2-weighted imaging. The axial ADC **(G)** and eADC **(H)** sequences demonstrate a dense, compact component with partial restricted diffusion. A 23-year-old with adenoid cystic carcinoma of the right lacrimal gland **(I–N)**. CT axial **(I)** and coronal **(J)** sequences demonstrate bony orbital destruction. MRI T1-weighted axial **(K)**, T1-weighted fat-suppressed contrast-enhanced coronal **(L)**, coronal STIR **(M)**, and T2-weighted fat-suppressed axial **(N)** sequences demonstrate a large, well-defined mass, that is isointense on T1-weighted imaging and hyperintense on T2-weighted imaging, relative to the extraocular muscles. Mild contrast enhancement is present.

On MRI, pleomorphic adenomas typically demonstrate hypo-isointense signal on T1-weighted imaging and iso-hyperintense signal on T2-weighted imaging when compared to surrounding muscle (1, 2, 10, 16, 19). Large lesions or those with haemorrhage, necrosis, or cystic changes, may show heterogeneous signals on both T1- and T2-weighted sequences (10, 19). A pseudocapsule may present as a thin, low intensity rim on both sequences (19). Gadolinium-enhanced MRI reveals moderate, often uniform enhancement (2, 3, 6, 16, 19). In a study of 35 patients, T1-weighted MRI demonstrated homogeneous signal in 86% and isointense signal to muscle in 97% of cases. T2-weighted images showed heterogeneous (91%) and high intensity (86%) signals, with moderate enhancement in 91% (23). Unlike malignant or some inflammatory lacrimal gland lesions, pleomorphic adenomas almost always demonstrate a rounded, well-circumscribed posterior margin, and the ‘wedge sign’ is typically absent (3, 6, 11, 18). This sign refers to posterior extension of a tumour along the lateral or superior orbital wall, following the contour of the bone and adjacent rectus muscles, without intraconal spread, often tapering to a point (**Figure 3**) (6, 11).

On US, pleomorphic adenomas appear well-defined with a highly reflective pseudocapsule on A-scan mode. They frequently contain multiple internal septations (6). There is usually a homogeneous internal appearance. Cystic areas appear hypoechoic on B-scan mode (11).

#### 3.1.2 Myoepithelioma

Formerly considered a variant of pleomorphic adenoma, myoepithelioma is now classified as a distinct entity composed almost exclusively of neoplastic myoepithelial cells (11). It is a rare lacrimal gland tumour, described only in case reports (24). Imaging findings often overlap with those of pleomorphic adenoma, making definitive preoperative differentiation challenging (1). Reported cases typically show a well-circumscribed mass, frequently associated with inferonasal displacement of the globe (19, 24, 25). There is no predilection for laterality (11). On CT, myoepitheliomas may appear as well-encapsulated lesions (19). MRI may show a thin layer of fat separating the tumour from both the superior and lateral rectus muscles (24). On T1-weighted imaging, the mass is isointense to muscle, while on T2-weighted sequences, it appears homogeneously hyperintense to muscle (24). Post-contrast imaging demonstrates marked enhancement (24).

#### 3.1.3 Warthin tumour

Warthin tumour of the lacrimal gland is a rare benign epithelial neoplasm, far more common in the parotid gland (19). It has been only sparsely reported in the literature, with a 1997 report describing a large, round, cystic-like mass in the right lacrimal fossa on CT imaging (26).

#### 3.1.4 Oncocytoma

Oncocytomas are benign epithelial tumours arising from proliferation of oncocytes, which result from metaplasia of ductal or acinar epithelial cells in the lacrimal gland (11). As lacrimal gland involvement is rare, imaging findings are not well established. Case reports describe an intraorbital extraconal mass in the lacrimal fossa on CT and MRI (27, 28). The mass may be cystic (19) and can cause inferonasal globe displacement (28). On MRI, it may appear hypointense to orbital fat on T1-weighted imaging, and hyperintense relative to orbital fat on T2-weighted imaging (27), usually without bone invasion (29).

#### 3.1.5 Cystadenoma

Cystadenoma is an exceedingly rare benign epithelial tumour of the lacrimal gland, thought to arise from ductal epithelial tissue (11, 30). A CT case report of bilateral lesions showed soft tissue masses with internal fluid attenuation and septations, without discrete visualisation of the lacrimal glands or bony erosion (11). B-scan ultrasonography demonstrated multi-cystic masses in the superolateral orbits (11).

### 3.2 Malignant

#### 3.2.1 Adenoid cystic carcinoma

Adenoid cystic carcinoma is the most common malignant epithelial tumour of the lacrimal gland, accounting for approximately 60% of all malignant lesions (1, 2, 9, 12). It is characterised by aggressive local invasion, high recurrence, and frequent perineural spread (1). Radiologically, it presents as a unilateral, solid, globular or nodular mass (19), usually arising from the orbital lobe of the lacrimal gland (2, 9). Margins are typically irregular (11, 12, 16, 17, 20) or ill-defined (16, 17), though some lesions may appear well-circumscribed (2, 3, 6, 11). Involvement of adjacent tissues and early bone destruction distinguish it from benign lesions (3, 6, 11, 16–18).

CT is valuable for assessing bone involvement, often showing lacrimal fossa expansion (19) and lytic destruction or sclerosis of adjacent bone (20) – both of which occur early in the disease course and are key features differentiating adenoid cystic carcinoma from benign lesions (**Figure 3**) (6). One study reported bone erosion in 75% of cases and bone destruction in 34% (31). Calcifications occur in approximately 20% of lesions but are non-specific and do not help in differentiating adenoid cystic carcinoma from other lacrimal gland tumours (9, 11, 19). Moderate contrast enhancement is typical (1–3, 10–12). High-resolution and bone-window images are recommended (19).

On MRI, the tumour exhibits variable signal intensities. Relative to muscle, the tumour appears hypointense to isointense on T1-weighted imaging (1, 2, 6, 16), and isointense to hyperintense on T2-weighted sequences (1, 2, 6, 16). Gadolinium enhancement is typical (1–3, 10–12). On T2-weighted sequences, areas of central necrosis appear hyperintense, while calcifications appear hypointense (11). Contrast-enhanced MRI is helpful for detecting perineural spread and invasion into the cavernous sinus, brain, or bone marrow (1–3, 6, 12, 19). Perineural invasion most commonly involves the optic nerve and ophthalmic division (V1) of the trigeminal nerve, correlating with poor prognosis (1, 2, 10). Dural enhancement may also occur (10, 19). Gradient echo MRI may reveal layered structures, indicative of the stepwise process of haemorrhagic expansions and degenerative changes within the tumour (6). The wedge sign is highly suggestive of malignancy (3), occurring laterally in 66.7% of cases and superiorly in 55.5%; the latter carries a 13.44-fold greater risk of malignancy compared to benign tumours (18).

On US, adenoid cystic carcinoma often appears more heterogeneous and echogenic than benign lesions (6). A-scan typically shows a regular internal structure with medium to high reflectivity,^9^ with occasional hyporeflective areas corresponding to cystic cavities seen on B-scan (11).

#### 3.2.2 Carcinoma ex pleomorphic adenoma

Carcinoma ex pleomorphic adenoma, also termed malignant mixed tumour, is the second most common malignant tumour of the lacrimal gland. It denotes malignancy of any histologic type arising within or from a primary (*de novo*) or recurrent pleomorphic adenoma (1, 11, 32). Malignant transformation, which can take 15–20 years, can occur as either a *non-invasive carcinoma* with focal malignant areas within the adenoma, or as an *invasive carcinoma* extending into surrounding tissues (1, 6).

Imaging features depend on invasiveness. Non-invasive carcinomas are typically well-circumscribed, with smooth, well-defined margins (19, 33) resembling benign pleomorphic adenomas, though histology will demonstrate features of malignancy without signs of local invasion (6). In contrast, invasive carcinomas are more aggressive (1) and demonstrate ill-defined (3) or irregular margins (6), heterogeneous density (1, 3, 6, 33, 34), areas of cystic degeneration, extra-orbital spread (3, 6), and bone remodelling, erosion (1, 3, 33) or destruction (1, 6, 11, 19). Additional features include intraconal extension, peripheral enhancement of necrotic areas, inferior globe displacement, and posterior extension through the superior orbital fissure (33, 34). In a series of five patients, bone erosion occurred in 100%, bone invasion in 60%, and intralesional calcification in 80% (31).

On MRI, the mass may show moderate contrast enhancement (33), which can help identify dural involvement and intracranial extension, if present (6). MRI signal characteristics may show low signal intensity on both T1- and T2-weighted sequences (33). In a study of 26 cases, findings included lacrimal fossa remodelling (77%), bone erosion or destruction (35%), cystic or multilobulated internal structures (27%), focal intralesional calcification (27%), and peripheral rim-enhancement (8%) (33).

#### 3.2.3 Mucoepidermoid carcinoma

Mucoepidermoid carcinoma is a rare malignant tumour of the lacrimal gland, representing approximately 1% of all lacrimal gland neoplasms (1, 2). Its radiological features resemble those of other malignant neoplasms, such as adenoid cystic carcinoma (2), with definitive diagnosis made histologically (1, 6). Imaging shows a heterogeneous, aggressive, and ill-defined mass with irregular margins (1, 35, 36). Rapid growth of this infiltrative lesion often causes bony scalloping or destruction, with occasional invasion into adjacent structures, including the brain (1, 2, 11, 35, 36). Additional findings include globe displacement (35, 36), cystic areas, and intralesional calcifications (2, 11, 36). On MRI, the mass demonstrates contrast enhancement (35), and may show high signal intensity on T1-weighted imaging due to its relatively high lipid content (6).

#### 3.2.4 Carcinosarcoma ex pleomorphic adenoma

The carcinosarcoma ex pleomorphic adenoma of the lacrimal gland is extremely rare, with only a few reported cases (37). In one, imaging showed a well-circumscribed lacrimal fossa mass with marked heterogeneous enhancement on MRI (38).

#### 3.2.5 Squamous cell carcinoma

Squamous cell carcinoma of the lacrimal gland is rare and may arise from heterotopic tissue, or more typically, spread from nearby structures, such as the upper eyelid (6) or conjunctiva (10). It may also result from malignant transformation of the epithelial wall of a benign cyst (e.g. dacryocyst) (6).

Imaging findings of primary lacrimal gland squamous cell carcinomas are limited to a couple of case reports. Similar to other malignant masses, squamous cell carcinomas often have ill-defined margins and infiltrate adjacent tissues (39, 40). Bony involvement can vary (39). CT imaging in one case report revealed an orbital mass without bone involvement (39), whilst another demonstrated extensive bony destruction to the frontal and zygomatic bones (40). MRI findings also vary. In one case, T1-weighted MRI demonstrated a lesion with low signal intensity, isointense to muscle or gland parenchyma, while T2-weighted imaging showed a heterogeneous lesion with areas of hyperintense signal. Gadolinium-enhanced MRI showed heterogeneous enhancement with small non-enhancing areas, possibly representing cystic change or necrosis (39). In contrast, another report demonstrated a heterogeneously hyperintense multi-lobulated lesion on T1-weighted imaging. T2-weighted imaging showed that the mass was mostly isointense to the brain (40).

#### 3.2.6 Oncocytic carcinoma

Oncocytic carcinoma of the lacrimal gland may develop *de novo*, or more commonly, through malignant transformation of a pre-existing benign oncocytoma (41). The tumour may be irregularly shaped (41), enhance with contrast (42), or extend intracranially (19, 27, 41). Like other malignant lacrimal gland lesions, it may demonstrate bone destruction (19, 41).

#### 3.2.7 Lymphoepithelial carcinoma

Radiological findings of lacrimal gland lymphoepithelial carcinoma are scarce. Orbital CT and MRI may reveal a well-defined lacrimal gland mass without bone involvement (43–45). On MRI, the lesion often appears homogeneous, with gadolinium enhancement (43, 44).

#### 3.2.8 Sebaceous carcinoma

Like squamous cell carcinoma, primary sebaceous carcinoma can rarely originate in the lacrimal gland due to heterotopic tissue (6, 19, 45). However, secondary invasion from eyelid sebaceous carcinoma (meibomian or Zeiss glands), is far more common, and can mimic a primary lacrimal gland tumour (6, 11, 45, 46). It is highly malignant and must be differentiated from locally invasive eyelid sebaceous carcinoma or a metastatic deposit (19). Furthermore, other lacrimal gland neoplasms can undergo sebaceous differentiation, potentially mimicking a primary sebaceous carcinoma (45).

Imaging findings resemble other malignant lacrimal gland tumours, typically showing a soft tissue mass with associated bony erosion (6, 47). Lesions may have well-defined borders (48, 49), mostly homogeneous signal (48, 49), contrast enhancement (49), and invasion of adjacent structures including the globe, optic canal, and cavernous sinus (47). On T1-weighted MRI, high signal intensity may be seen due to its high lipid content, similar to mucoepidermoid carcinoma (6).

#### 3.2.9 Ductal adenocarcinoma

Primary ductal adenocarcinoma is an increasingly recognised subtype of lacrimal gland adenocarcinoma (50, 51). Though rare, cases are increasing. It demonstrates aggressive behaviour similar to salivary duct carcinoma, including perineural and vascular invasion, local recurrence, and metastasis (45, 52).

Imaging commonly shows an irregular, infiltrative mass with ill-defined margins (11, 50–54) and heterogeneous attenuation (52, 55). The globe may be displaced inferolaterally (54, 56). Local infiltration may extend into the intraconal space (54, 56) and involve adjacent extraocular muscles (51, 53, 54). Bone erosion (57), destruction (11, 17), or remodelling (11, 56) may be extensive, commonly affecting the supraorbital and lateral orbital walls, and less frequently the medial orbital wall, inferior orbital wall, greater wing of the sphenoid, sphenoid sinus, basilar clivus, and frontal bone. Peripheral tissue invasion may involve adipose tissue, levator palpebrae, superior rectus, or eyelid (58). Calcifications may occur (53), and contrast enhancement is typical on CT (55, 56) and MRI (50, 52).

On MRI, one case showed the supraorbital nerve encased within the mass, exhibiting abnormal enhancement, along with infiltration of the preseptal soft tissues extending over the lateral orbital rim (54). Another case showed a hyperintense lesion on T1-weighted sequences, and a slightly hypointense signal on T2-weighted sequences compared to extraocular muscles, with local tissue invasion (58). In one patient with neurofibromatosis, US demonstrated a heterogeneous mass with variable reflectivity in both the intraconal and extraconal spaces, with the optic nerve shadow appearing separate from the lesion anteriorly (56).

In a review of 22 cases of primary lacrimal gland ductal adenocarcinoma, bony erosions occurred in 41% of cases, muscle involvement in 18%, and calcification in 9% (52). Another series of 25 lacrimal gland adenocarcinomas (19 ductal) reported bone destruction in 52% of cases, peripheral tissue invasion in 44%, and optic nerve or perineural invasion in 20% (58).

#### 3.2.10 Adenocarcinoma

Adenocarcinoma, not otherwise specified, is a carcinoma with glandular or ductal differentiation, but lacks distinctive histomorphological features of specific subtypes (16). Though rare, the tumour is the third most common primary malignant epithelial tumour of the lacrimal gland, comprising 5-10% of such neoplasms (11, 17). Differentiating adenocarcinoma from other neoplasms, such as polymorphous low-grade adenocarcinoma or mucoepidermoid carcinoma (grades 1 and 2), is essential, as these other tumours generally carry a better prognosis (32). Radiological findings are limited and may be underreported or misclassified.

#### 3.2.11 Polymorphous low-grade adenocarcinoma

Polymorphous low-grade adenocarcinoma is rare, representing only 1.1% of all lacrimal gland epithelial tumours in a review of 272 cases (59). One CT case report revealed an ill-defined mass with central radiolucency, rim enhancement, and no bony involvement (60).

#### 3.2.12 Basal cell adenocarcinoma

Basal cell adenocarcinoma is a rare epithelial neoplasm sharing cytological characteristics with basal cell adenoma, but is distinguishable by its infiltrative nature and extremely low metastatic potential (45). CT imaging in one case report demonstrated a homogeneous lacrimal gland mass with smooth borders and no bone erosion (61).

#### 3.2.13 Cystadenocarcinoma

Cystadenocarcinoma is a rare lacrimal gland neoplasm, histopathologically characterised by numerous cysts, often with a predominant papillary component. One case report revealed an irregular, circumscribed, multi-cystic mass with small foci of calcifications on CT and MRI (45). In another report, CT imaging showed a homogeneous, ovoid-shaped, extraconal tumour located in the right lacrimal fossa without bone involvement, along with several intra-lesional calcifications. MRI demonstrated two large high-intensity cystic lesions and several smaller ones, occupying most of the tumour on T2-weighted imaging (62).

#### 3.2.14 Neuroendocrine carcinoma

Neuroendocrine tumours of the lacrimal gland are extremely rare, originating from neuroendocrine cells of the ‘diffuse endocrine system’. All documented cases are high-grade malignancies (17),and no radiological findings were identified.

#### 3.2.15 Merkel cell carcinoma

Merkel cell carcinoma is an aggressive cutaneous neuroendocrine carcinoma, with 5-10% of cases occurring in the eyelids, likely due to sun exposure. It is extremely rare in the lacrimal gland (63), with limited cases reported, and no radiological data available.

#### 3.2.16 Acinic cell carcinoma

Acinic cell carcinoma of the lacrimal gland is extremely rare, more frequently reported in the parotid gland, where it accounts for 2-4% of neoplasms (64, 65). The lesion has a propensity for local recurrence and distant metastases.

Radiologically, these tumours present non-specifically with a benign appearance, often resembling pleomorphic adenomas (65). Case reports have described CT findings of a well-defined, well-circumscribed, ovoid mass with heterogeneous density, or a central region of low attenuation (65). It can contain mixed cystic and solid components (64), and show contrast enhancement. The tumour may show calcifications and cause inferior globe displacement or bony changes, including scalloping or destruction (64–66). The greater wing of the sphenoid, along with the superior, posterior, and lateral orbital walls, may also be involved (64, 66). Exophthalmos has been reported, with one case demonstrating a displacement of 3.5mm compared to the unaffected orbit (65).

On MRI, the T1-weighted spin-echo sequence can effectively delineate the separation between the mass and the lateral rectus muscle (65). On T2-weighted sequences, the mass may exhibit intermediate signal intensity with enhancement (66).

#### 3.2.17 Myoepithelial carcinoma

Myoepithelial carcinoma of the lacrimal gland is rarely documented. The tumour may demonstrate variable margins, enhancement, inferior globe displacement, bone destruction, and invasion of surrounding structures such as the lateral rectus muscle (10, 67, 68). MRI may exhibit non-specific low T1- and high T2-weighted signal intensities (10).

#### 3.2.18 Epithelial-myoepithelial carcinoma

The epithelial-myoepithelial carcinoma is an uncommon, low-grade malignant epithelial neoplasm characterised by a biphasic composition, consisting of both myoepithelial and ductal cells (45). It can arise through several pathways. In a review of 11 patients with lacrimal gland involvement, five (45.4%) arose from a pre-existing pleomorphic adenoma, five were *de novo*, and one from an unknown variant (69). Tumours may appear lobulated with well-circumscribed margins, heterogeneous density, and contrast enhancement (70–72). There is commonly inferior globe displacement, calcifications, and no bony erosion (68, 70–73). Advanced cases may cause bone expansion, thinning, remodelling, or local invasion, including the frontal process of the zygomatic bone (69). On MRI, it may appear as a low intensity mass on T1-weighted imaging and a high intensity mass on T2-weighted images (71).

#### 3.2.19 Adenosquamous carcinoma

Adenosquamous carcinoma is an extremely rare malignant tumour, characterised histologically by the presence of both glandular and squamous components; the latter arising from ductal epithelium or squamous metaplasia. One CT case report revealed a large intraorbital mass with calcifications and no intracranial extension (74).

#### 3.2.20 Large cell carcinoma

Large cell carcinoma of the lacrimal gland is exceedingly rare, with potentially only two reported cases. CT may reveal a heterogeneous, enhancing, irregularly shaped mass, likely accompanied with bone erosion and infiltration of extraocular muscles (75, 76). T2-weighted sequences revealed a high-intensity, irregular mass with infiltration of extraocular muscles in a single case report (75, 76).

#### 3.2.21 Intraductal carcinoma of the lacrimal gland

Intraductal carcinoma, primarily described in salivary glands, is exceedingly rare but has also been reported in the lacrimal gland as a low-grade variant. One CT case report demonstrated a well-defined, solid, enhancing mass without bony erosion or excavation (77).

#### 3.2.22 Metastases

Orbital metastases are rare, typically affecting the extraocular muscles and orbital fat (78). Isolated lacrimal gland metastases are even less common, presenting as either gland enlargement or a discrete mass, occasionally accompanied with orbital bony changes (1, 3, 78). These metastases typically arise via haematogenous spread from distant primaries (78). Compared to metastasis, direct extension from tumours in adjacent structures is far more common (1).

In adults, the most frequent primary cancers metastasising to the lacrimal gland include breast, hepatocellular, and renal cell carcinoma. Other reported primaries include renal, mediastinal, and ileocaecal carcinoids, lung neuroendocrine carcinoma, anorectal squamous cell carcinoma, gestational choriocarcinoma, adrenocortical carcinoma, colorectal adenocarcinoma, endometrial carcinosarcoma, pleural mesothelioma, thyroid carcinoma, tongue/tonsil squamous cell carcinoma, and oesophageal squamous cell carcinoma (1–3, 78). In children, Ewing sarcoma or neuroblastoma more commonly involve the orbit, often extending from bone to secondarily involve the lacrimal gland (1, 2). Hepatocellular carcinoma tends to present with orbital bony changes and intracranial extension. Breast cancer more commonly causes isolated lacrimal gland enlargement without associated bony changes. Renal cell carcinoma may present with either pattern (78).

Imaging features are non-specific and often overlap with primary malignant lacrimal gland neoplasms (1, 2). Lesions are typically unilateral, predominantly involving the orbital lobe (1). Bone involvement, including thinning, erosion, osteolysis, and notching, may occur (2, 78). Metastatic lesions usually show intermediate to high signal intensities on both T1- and T2-weighted images, along with heterogeneous contrast (78). Adjacent structures, including the eyelid and extraocular muscles, may be involved, and intracranial extension occurs in 57.1% of cases with bone involvement (78).

## 4 Lymphoid tumours

### 4.1 Benign

#### 4.1.1 Reactive lymphocytic hyperplasia

The lacrimal gland contains native lymphoid tissue, which can give rise to lymphoid tumours (2, 6). However, only about 20% of orbital lymphoproliferative diseases originate in the lacrimal gland. The majority of these cases are thought to result from secondary lymphoid involvement from extra-orbital sources (6). Lymphoid tumours encompass a wide spectrum, from benign lymphoid hyperplasia to highly aggressive lymphomas (10). Lymphoid hyperplasia, or pseudolymphoma, is classified as either ‘reactive’ or ‘atypical’ – the former is entirely benign in morphology and immunophenotype, while the latter represents borderline lesions difficult to distinguish from lymphoma. Retrospective studies have shown that many cases classified as ‘atypical’ were ultimately found to be low-grade non-Hodgkin lymphomas (79). Although lymphoid tumours vary in type, they often appear similar on current imaging modalities, making tissue biopsy often necessary for accurate diagnosis (6, 79).

Lymphoid hyperplasia usually involves the lacrimal glands bilaterally, involving both orbital and palpebral lobes (1, 2, 19), whereas epithelial tumours typically affect only one (79). Compared to lymphoma, lymphoid hyperplasia demonstrates greater heterogeneity, particularly on post-contrast MRI (1) and CT (2, 6). As with all lymphoid tumours, these lesions usually appear as rounded or lobulated masses with concave inner margins, diffuse contrast enhancement, and sharply defined borders against bone or sclera; however, interfaces with muscle or retrobulbar fat may appear streaky or serrated (2, 79). They often mould to adjacent orbital structures such as the globe and bony walls, with minimal displacement or indentation (1, 19, 79) Osseous erosion is rare (79). Smooth expansion of the lacrimal fossa, though uncommon, should raise suspicion for epithelial or more aggressive pathology (79). Mild enlargement of adjacent EOMs may occur.

On CT, lymphoid hyperplasia is typically isodense to muscle (79). On MRI, it appears isointense on T1-weighted sequences and hypointense-isointense on T2-weighted sequences, relative to muscle, with an increased FLAIR signal, likely due to the lipid-rich membranes of densely packed lymphocytes (1, 2, 79). In our case, there was evidence of a restricted diffusion on DWI. A characteristic ‘wedge sign’ may be observed, with the lateral wedge sign present in 28.6% of reactive lymphoid hyperplasias and 35.7% of lymphomas, and the superior wedge sign in 7.1% and 21.4%, respectively (18).

#### 4.1.2 Angiolymphoid hyperplasia with eosinophilia/Kimura’s disease/Epithelioid haemangioma

Angiolymphoid hyperplasia with eosinophilia, or epithelioid haemangioma, is a rare clinicopathologic condition that shares both clinical and histopathologic features with Kimura disease (80, 81). Although the two are often used interchangeably, they represent distinct entities (80, 81). Given the overlapping characteristics, these conditions will be discussed together, as their distinction in the existing literature remains unclear. These conditions are characterised by inflammatory angiomas with eosinophilic infiltration, primarily affecting the skin of the head and neck (6).

**Figure 4:**
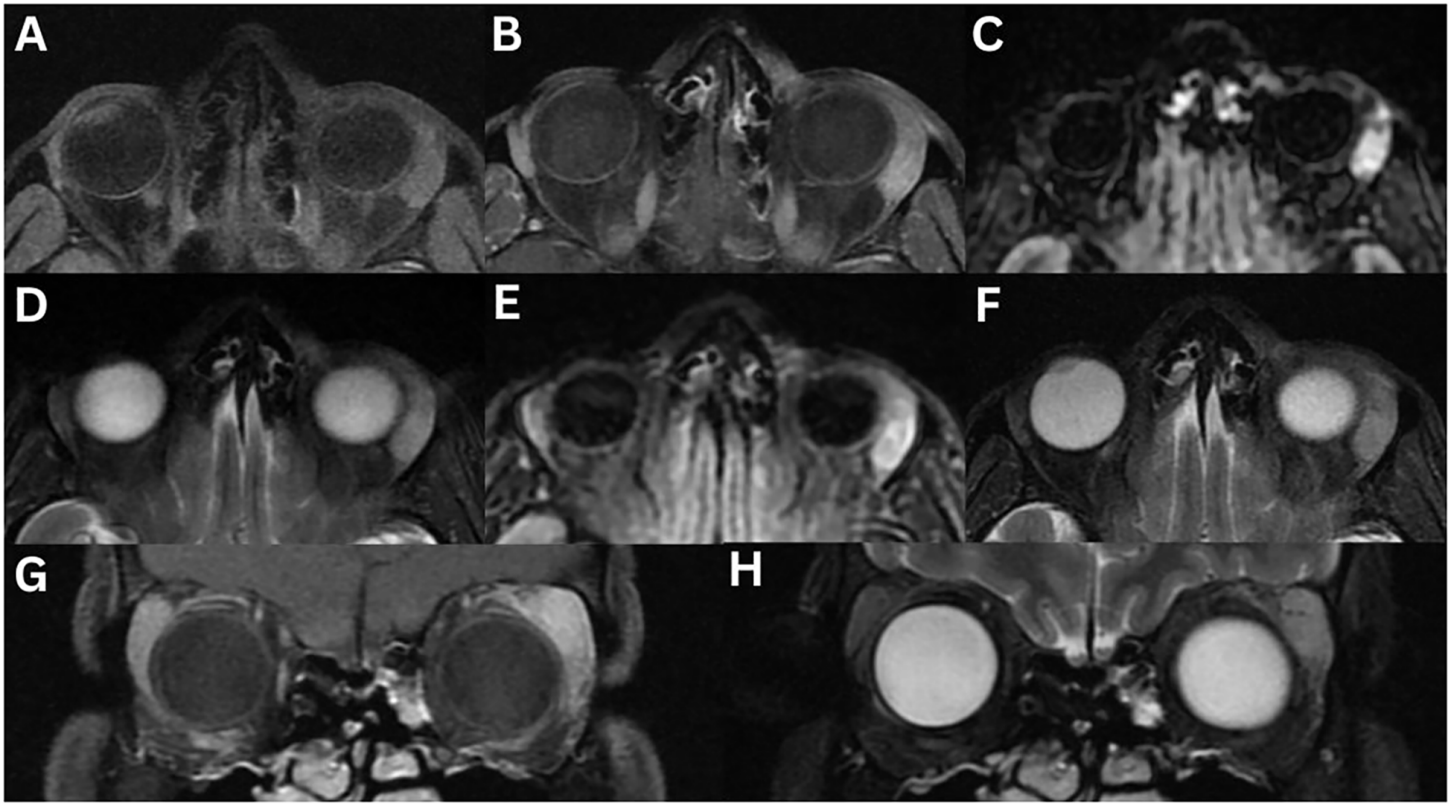
Angiolymphoid hyperplasia with eosinophilia of the left lacrimal gland. MRI T1-weighted axial fat-suppressed FLAIR **(A)** and axial fat-suppressed contrast-enhanced **(B)**, T2-weighted axial DIR **(C)**, axial **(D)**, axial fat-suppressed FLAIR **(E)** and axial fat-suppressed **(F)**, T1-weighted coronal fat-suppressed contrast-enhanced **(G)**, and T2-weighted coronal fat-suppressed **(H)** sequences demonstrate a well-defined, solid, homogeneous mass lesion involving the left lacrimal gland. The mass is hypointense on T1-weighted imaging and hyperintense on T2-weighted and FLAIR sequences, relative to extraocular muscles. There is moderate homogeneous enhancement post-contrast.

Orbital, eyelid, and lacrimal gland involvement is uncommon (81). CT findings from limited case reports show a well-defined, solid, homogeneous swelling involving both the palpebral and orbital lobes of the lacrimal gland, with diffuse post-contrast enhancement and no bony changes. Adjacent anterior orbital structures, including the canthus, eyelids, preseptal skin and fascia, extraconal fat, and extraocular muscles, may also be involved (6, 80–82). In one of our cases, MRI demonstrated a lesion isointense to muscle on both T1- and T2-weighted imaging, without bony involvement (**Figure 4**). In another, US revealed a lesion with medium-low to medium reflectivity, posterior high sound attenuation, and a slightly irregular internal structure (81).

### 4.2 Malignant

#### 4.2.1 Lymphoma

Lacrimal gland lymphomas encompass various subtypes, including extranodal marginal zone lymphoma, diffuse large B-cell lymphoma, follicular lymphoma, mantle cell lymphoma, chronic lymphocytic leukaemia or small lymphocytic lymphoma, and unclassified B-cell lymphoma (11). These subtypes share similar appearances and cannot be differentiated by imaging criteria alone (2). Additionally, differentiating lymphoma from reactive lymphoid hyperplasia is often difficult and may require serial imaging to assess growth patterns (6).

Lacrimal gland lymphomas are often bilateral, well-defined, and oblong, with homogeneous density. Unlike epithelial tumours, they typically involve both palpebral and orbital lobes diffusely (1–3, 6, 10–12). In a study of 16 patients, bilateral disease occurred in six (37.5%) (83). Palpebral lobe involvement often causes anterior expansion beyond the orbital rim (6). The tumour may envelop the globe, producing a concave ‘pancake-like’ appearance (10). Calcification is rare, and inferomedial globe displacement can occur (6). Enlargement is significant, and typically greater than in thyroid eye disease, granulomatosis with polyangiitis, sarcoidosis, IgG4-related ophthalmic disease, and non-specific dacryoadenitis (83–85).

**Figure 5:**
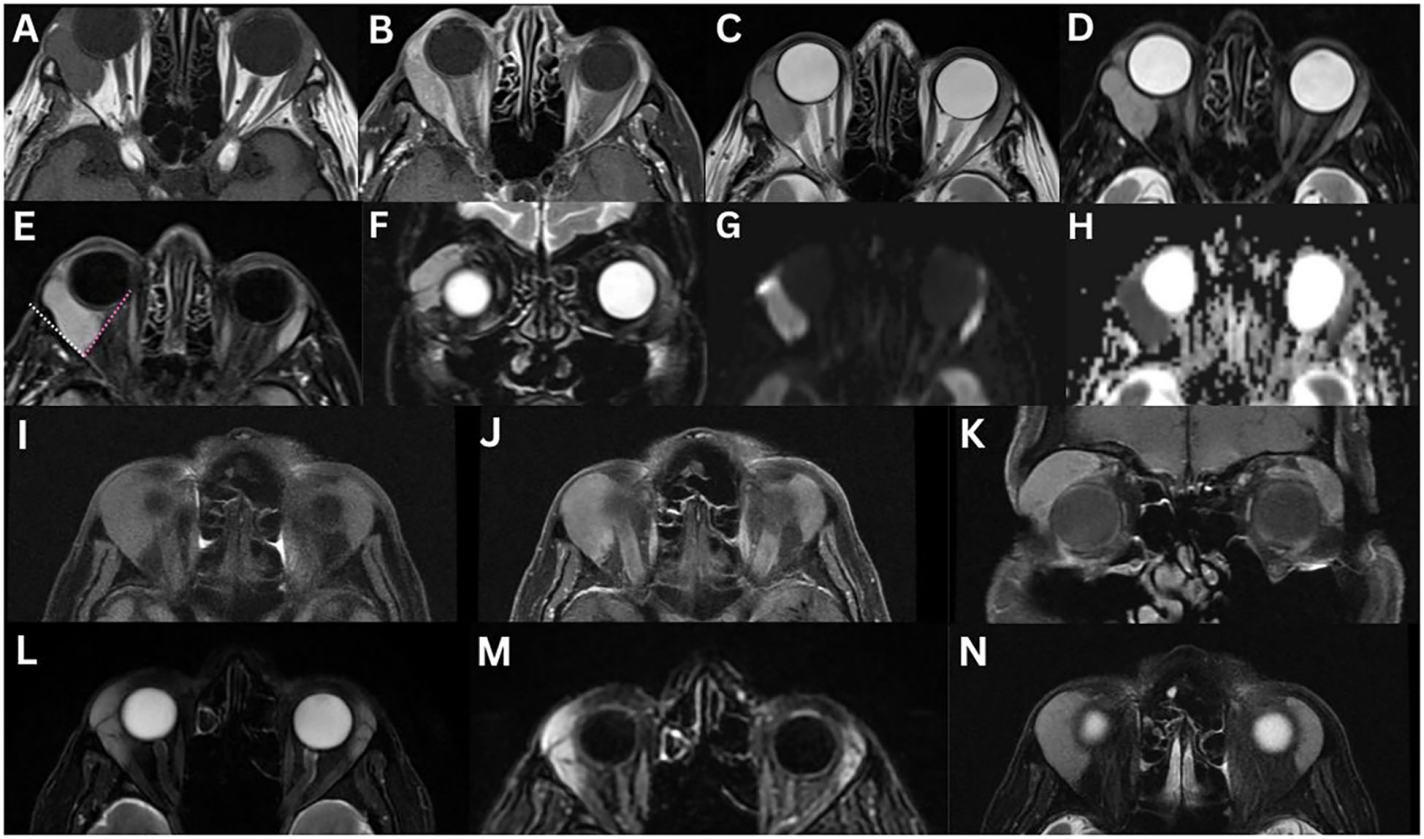
Marginal lymphoma of the right lacrimal gland **(A–H)**. MRI T1-weighted axial **(A)**, axial fat-suppressed contrast-enhanced **(B)**, T2-weighted axial **(C)**, axial fat-suppressed **(D)**, axial FLAIR **(E)**, and T2-weighted coronal fat-suppressed **(F)** sequences demonstrate a well-defined homogeneous mass that is isointense on T1-weighted imaging, and hyperintense on T2-weighted imaging, relative to extraocular muscles. The mass moderately enhances following contrast administration. There is evidence of a lateral wedge sign **(B)**. The posterior angle between the lacrimal gland (pink dotted line) and lateral orbital wall (white dotted line) is acute **(E)**. There is restricted diffusion on axial DWI **(G)** and ADC **(H)**. Mantle cell lymphoma involving the lacrimal glands **(I–N)**. MRI T1-weighted axial fat-suppressed FLAIR **(I)** and fat-suppressed contrast-enhanced axial **(J)** and coronal **(K)**, T2-weighted axial **(L)**, axial fat-suppressed FLAIR **(M)**, and axial T2-weighted fat-suppressed **(N)** sequences demonstrate asymmetrical bilateral enlargement of the lacrimal glands, more pronounced on the right. The right lacrimal gland, confirmed by biopsy to be mantle cell lymphoma, demonstrates a homogeneous intermediate intensity signal on T1-weighted imaging and a high intensity signal on T2-weighted imaging. There is homogeneous enhancement with contrast administration.

Lacrimal gland lymphomas typically demonstrate mild-to-moderate enhancement on CT and moderate-to-high enhancement on MRI (**Figure 5**) (1–3, 6, 9, 10). The tumour often conforms to the globe and adjacent bone, with associated bony remodelling such as scalloping or sclerosis (1, 2, 6, 12). Generally, there is no infiltrative bone destruction or indentation (2, 11, 83); however, osseous erosion may occur with diffuse large B-cell lymphoma (1). The tumour may also extend beyond the lacrimal gland, being reported in three out of five lymphoma cases in one study (85). It may infiltrate through the superior or inferior orbital fissures into the structures beyond (6). When the tumour spreads into potential spaces between fascial planes, its growth may become less well-defined (6).

CT typically shows an isodense (2, 6) to hyperdense (1) lesion, relative to muscle. On T1-weighted MRI sequences, the mass is isointense to muscle (1, 2, 6, 9, 12). T2-weighted sequence findings are variable (1, 2, 6, 9). DWI imaging reveals restricted diffusion with low ADC values, reflecting high cellularity (1, 2, 9, 84). The lower ADC value can serve as a reliable indicator to help distinguish lymphoma from benign lesions (84). Dynamic contrast-enhanced MRI shows enhancement in the early phase, followed by washout in the delayed phase (83).

In one series (n = 16), the wedge sign appeared in 87.5% of cases (83), while another reported 57.1% (n = 14) (18). The posterior angle between the lacrimal gland and lateral orbital wall was obtuse in 93.8% and acute in 6.25% (**Figure 5**) (83). Heterogeneity appeared on CT in 8.3% of cases, on T2-weighted imaging in 33.3%, and on contrast-enhanced T1-weighted imaging in 27.3% (83). Contrast enhancement was high in 83.3% of cases on CT, and in all cases on MRI (83). T2-weighted signal intensities were isointense in 75% of cases and hypointense in 25%, relative to grey matter; however, these findings conflict with other studies (83). Dynamic contrast-enhanced patterns showed washout (50%), plateau (25%), and prolonged (25%) (83). Extra-orbital spread involved the inferior orbital nerve (6.3%), salivary glands (20%), nasopharynx (6.3%), and lymph nodes (12.5%) (83).

### 4.3 Leukaemic lesions

#### 4.3.1 Leukaemia-chloroma or granulocytic sarcoma

Orbital granulocytic sarcoma, also referred to as leukaemia-chloroma due to its characteristic green colour from high myeloperoxidase content, can rarely involve the lacrimal gland secondarily. It represents a localised, extramedullary tumour of myeloid-origin cells (2, 10). Ophthalmic signs may be the initial manifestation of the disease (1), primarily involving the uvea, choroid, retina, and optic nerves, with secondary extension to one or both lacrimal glands (10).

Imaging may reveal a homogeneous, well-defined, infiltrative soft tissue mass involving the lacrimal gland, with associated soft tissue and bony lytic changes (1, 2). Thickening of the ocular coats and optic nerve are important diagnostic indicators (1). The lesion shows heterogeneous contrast enhancement (1, 2). Marrow involvement is common and best appreciated on MRI (2). On CT, the lesion appears hypodense to isodense relative to muscle (2). On MRI, it is usually hypointense to isointense on T1-weighted imaging relative to muscle (1, 2, 10). On T2-weighted imaging, the signal intensity is variable, ranging from hypointense (1) to hyperintense (10) relative to muscle, or isointense to brain cortex (2).

## 5 Mesenchymal tumours

### 5.1 Benign

#### 5.1.1 Granular cell tumour

Granular cell tumours have been reported throughout the body, but are rare in the orbit and ocular adnexa, with only one case involving the lacrimal gland (86). In that case, MRI revealed a well-circumscribed, homogeneous tumour with high post-contrast signal intensity (86).

#### 5.1.2 Fibrous histiocytoma

Fibrous histiocytoma, a common primary mesenchymal orbital tumour, consists of variable portions of fibroblasts and histiocytes (87). It has been reported in ocular and adnexal tissues, including the eyelids, conjunctiva, lacrimal sac, corneoscleral limbus and orbital tissue, with only one reported case involving the lacrimal gland (87). CT revealed a well-defined, isodense mass that remodelled the lacrimal fossa and indented the lateral aspect of the globe, without associated bone destruction. US showed a well-defined superotemporal mass with low internal reflectivity (87).

#### 5.1.3 Solitary fibrous tumour (hemangiopericytoma)

Orbital solitary fibrous tumour has recently been proposed as an umbrella term for both hemangiopericytoma and fibrous histiocytoma of the orbit (88). These are rare vascular tumours arising from the pericytes of Zimmermann, which normally surround the capillaries and postcapillary venules in tissues (10). Over 90 orbital cases have been reported (89), though lacrimal gland involvement is rare (89). Malignant transformation and, rarely, metastases may occur (10, 17).

Radiologically, these tumours are non-specific (90), appearing as a well-demarcated, ovoid, or nodular mass with regular margins, marked contrast enhancement, and no invasion of adjacent structures or bone remodelling (89–91). However, local invasion and bone involvement can occur (10, 19, 92). Signal intensity and heterogeneity vary depending on the tumour’s cellular composition, collagen content, and the presence of degenerative changes, necrosis, or haemorrhage (89, 90). Cystic changes may also be observed (89, 91).

CT may demonstrate a soft tissue density mass (90). MRI features are variable; on T1-weighted imaging, the mass may appear isointense relative to the surrounding extraocular muscles, while T2-weighted signals can vary, with the mass potentially appearing hypointense, isointense, or even hyperintense to muscle (89, 90). Additionally, angiography can help visualise a hypervascularised tumour, resembling an angioma (89).

#### 5.1.4 Neurofibroma (benign peripheral nerve sheath tumour)

Neurofibromas arise from the neural tissue of the lacrimal gland and surrounding structures, and can be classified as localised, diffuse, or plexiform. The latter is uncommon and almost exclusively pathognomonic of neurofibromatosis Type 1 (6, 9). Neurofibromas extending from the orbit typically originate from the orbital nerve sheath, while those involving the lacrimal gland itself likely arise from the gland’s own innervation (6).

Localised neurofibromas appear as well-circumscribed, homogeneous masses that may excavate the lacrimal fossa (6). These localised neurofibromas are generally less vascular than diffuse or plexiform neurofibromas, which tend to be more locally infiltrative and do not usually involve the lacrimal gland (6).

Lacrimal gland plexiform neurofibromas present as poorly delineated, potentially lobulated masses that infiltrate surrounding structures diffusely. They may involve craniofacial structures, causing bone erosion, thinning, or foraminal extension (9, 10, 93). These lesions may appear homogeneous (93) with moderate contrast enhancement (10), and demonstrate low signal intensity on T1-weighted imaging, and high signal intensity on T2-weighted imaging (10). Exophthalmos may occur (94). There was no evidence of restricted diffusion on DWI in one case report (95).

#### 5.1.5 Schwannoma (benign peripheral nerve sheath tumour)

Schwannoma is a benign neural tumour composed entirely of Schwann cells and may rarely affect the lacrimal gland. Like neurofibromas, Schwannomas may arise anywhere along the course of a peripheral sympathetic or cranial nerve, including the orbital nerve sheath, or originate directly from the gland’s own innervation (6, 96). Imaging may reveal a localised, well-defined, encapsulated, homogeneous or heterogeneous mass causing mass effect without invasion (1, 96). Bony remodelling and moulding around the globe have been reported (97), and intralesional calcification can occur (96, 98).

### 5.2 Malignant

#### 5.2.1 Synovial sarcoma

Synovial sarcoma is a rare, malignant soft tissue tumour, more common in adolescents and young adults (99, 100). Two cases have been reported in the lacrimal gland. CT imaging revealed a soft tissue density mass in the retroconal space, with solid and cystic components, irregular margins, mild enhancement, and associated proptosis, along with erosion of the lateral orbital wall and roof. No calcifications were present (99, 100).

#### 5.2.2 Malignant peripheral nerve sheath tumour

Malignant peripheral nerve sheath tumours are rare, highly aggressive neoplasms originating from peripheral nerves or their endings in soft tissues (101). Only isolated case reports describe orbital or periorbital involvement, with a single case documented within the lacrimal gland (101). CT imaging revealed a well-encapsulated, homogeneous, intermediate density mass causing indentation and inferonasal globe displacement, with associated bony erosion and excavation of the zygomatic bone. MRI showed a well-defined mass with intermediate T1- and high T2-signal intensity, with marked contrast enhancement (101).

General MRI findings of malignant peripheral nerve sheath tumours include a well-defined mass with mixed solid and cystic components. Perineural invasion may be indicated by nerve enlargement or neural enhancement on post-contrast images (101).

#### 5.2.3 Rhabdomyosarcoma

Rhabdomyosarcoma is an aggressive malignant mesenchymal tumour characterised by cells exhibiting histologic features of striated muscle at various stages of embryonic development (1, 102). It can arise throughout the body, including the ocular region. While most ocular rhabdomyosarcomas originate in orbital soft tissues, lacrimal gland involvement is rare. When present, the tumour may spread locally into adjacent structures such as the sinuses, nasal cavity, or intracranial cavity (6). In one reported case, MRI demonstrated a large, low-intensity soft-tissue mass in the right lacrimal fossa, associated with proptosis and extension into the surrounding periorbital soft tissues (6).

## 6 Dacryoadenitis

Dacryoadenitis, an inflammation of the lacrimal gland, can be acute or chronic. Chronic dacryoadenitis is often linked to underlying conditions, though many remain idiopathic (1). Distinguishing chronic dacryoadenitis from infiltrative tumours on imaging can be challenging (12). Radiologically, dacryoadenitis may present with diffuse unilateral or bilateral gland enlargement, with irregular margins and intense homogeneous contrast enhancement (3, 6). Similar to lymphoma, inflammatory lacrimal disease spreads along the orbital tissue planes, conforming to surrounding structures without bony erosion or globe distortion (3, 6).

Imaging may reveal diffuse enhancement of the sclera, extraocular muscles, orbital fat, or optic nerve sheath, depending on the extent of involvement (3). On MRI, the lacrimal gland may be isointense on T1-weighted imaging and hypointense on T2-weighted imaging, relative to muscle (6). US may demonstrate increased internal echogenicity, an echolucent area adjacent to the sclera, and anterior thickening of the lateral rectus muscle (6).

### 6.1 Idiopathic dacryoadenitis

Dacryoadenitis has a broad differential, including both inflammatory and infectious causes (83, 103). If no specific aetiology is identified, the condition is classified as idiopathic, or non-specific dacryoadenitis (83, 104). It represents the most frequent pathology found in lacrimal gland biopsies, comprising approximately 32.8% of cases (105), with some series documenting rates up to 78% (104). Since chronic dacryoadenitis is most commonly idiopathic and typically not further specified, it will be discussed in this context (1).

Inflammation may be confined to the gland or extend to surrounding structures, including the extraocular muscles, fat, and episcleral tissue (104). Bilateral enlargement occurs in up to 69% of cases, with ill-defined borders seen in 64% (83, 85, 104). Enlargement may be symmetric or asymmetric, involving both orbital and palpebral lobes (1, 2, 10, 85). Despite the enlargement, the overall shape of the gland is typically preserved (1, 85). CT usually shows no compressive changes or bony erosion, except in the sclerosing variant – many of which are now recognised as IgG4-related ophthalmic disease (1, 104, 106). MRI is the investigation of choice (104). The gland is isointense to muscle on T1- and T2-weighted imaging (104), with homogeneous, intense enhancement, and possible lateral rectus involvement. Similar findings can be observed on CT (1, 10, 83, 104).

In a series of 29 dacryoadenitis cases, 14% exhibited an associated mass or infiltration, 7% had muscle cone involvement, and none showed bone involvement (107). Other studies involving non-specific dacryoadenitis demonstrated globe indentation in 9.1% of cases (85), and a positive wedge sign in 54.8%, with the lacrimal gland-orbital wall angle being acute in 23.8% and obtuse in 76.2% (83). CT heterogeneity was observed in 21.2%, while MRI showed heterogeneity in 25% on T2-weighted imaging and 10.5% on contrast-enhanced T1-weighted sequences. Enhancement was high on CT in 97% and high on MRI in 94.7%. T2-weighted signal intensity was isointense in 85% of cases and hypointense in 15%, relative to grey matter. Dynamic contrast-enhanced pattern revealed washout in 71.4% of cases, plateau in 14.3%, and prolonged enhancement in 14.3%. Extra-orbital involvement was noted in the infraorbital nerve (4.8%, paranasal sinus (25%), salivary gland (20.7%), nasopharynx (9.5%) and lymph nodes (37.5%) (83).

### 6.2 Systemic inflammatory disease

#### 6.2.1 Idiopathic orbital inflammation (orbital pseudotumour)

Idiopathic orbital inflammation encompasses a heterogeneous group of conditions characterised by orbital inflammation without an identifiable local or systemic cause (6, 108). It can affect any orbital structure, either locally or diffusely (108). Localised forms may involve the extraocular muscles (orbital myositis), sclera (scleritis), uvea (uveitis), superior orbital fissure, cavernous sinus, or lacrimal gland (dacryoadenitis) (109). In diffuse cases, it can involve the orbital fatty tissues (109). Diagnosis is based on the exclusion of other aetiologies, including inflammatory, autoimmune and neoplastic conditions (2, 3, 108, 109).

**Figure 6:**
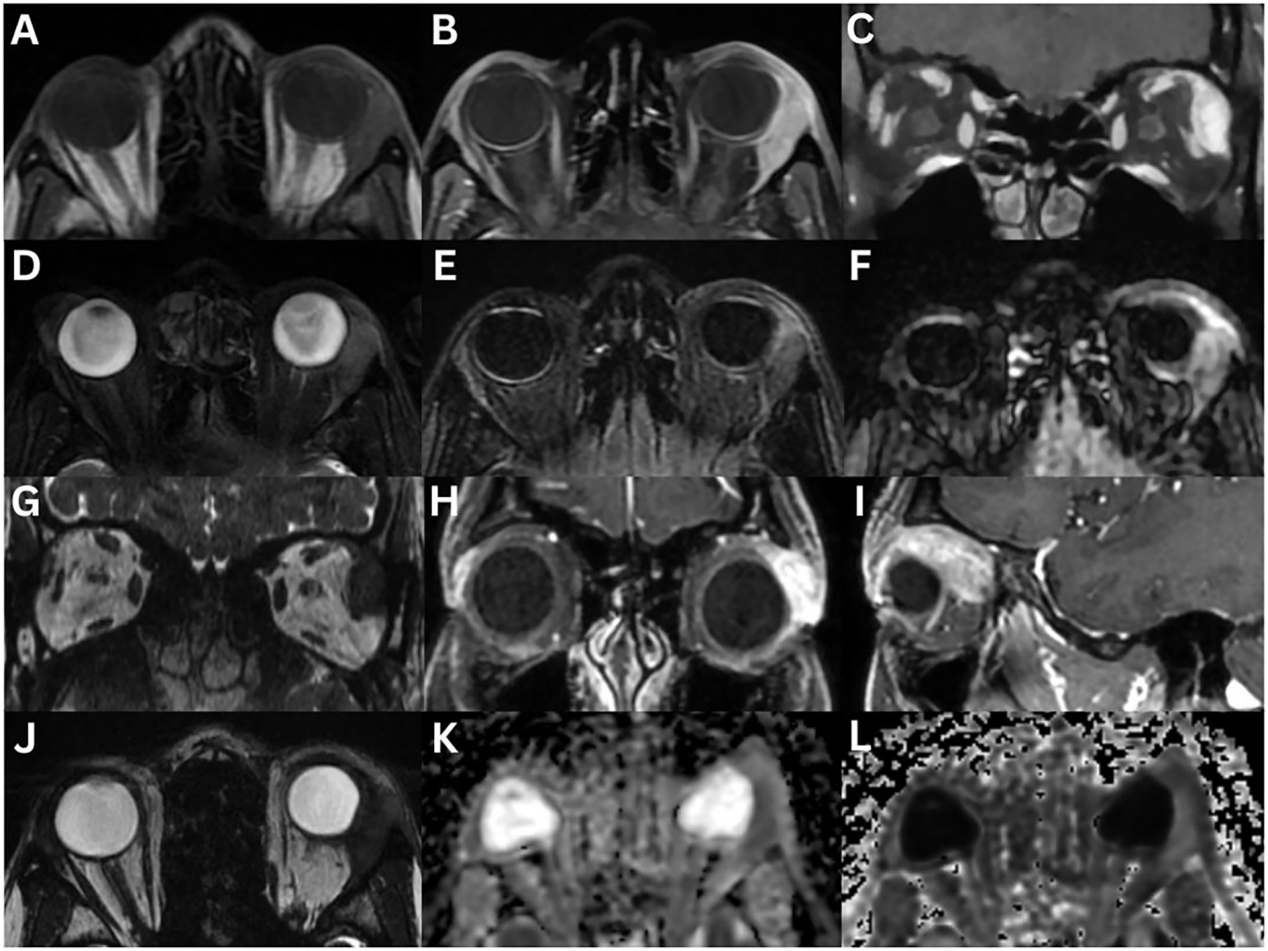
Idiopathic orbital inflammation of the left orbit. MRI T1-weighted axial **(A)**, axial fat-suppressed contrast-enhanced **(B)**, and coronal fat-suppressed contrast-enhanced **(C)**, T2-weighted axial **(D)**, axial fat-suppressed FLAIR **(E)**, axial 3D DIR CUBE **(F)**, coronal FIESTA **(G)**, coronal contrast-enhanced **(H)**, and sagittal contrast-enhanced **(I)** demonstrates enlargement of the left lacrimal gland. The lesion is homogeneous and solid, with intermediate signal intensity on T1- and T2-weighted images, and demonstrates marked contrast enhancement. Axial 3D Fiesta-C is also shown **(J)**. No restricted diffusion was observed on axial ADC **(K)** or eADC **(L)**.

The lacrimal gland may appear diffusely enlarged, unilaterally or bilaterally, involving both lobes while maintaining its overall shape (2, 109, 110). Periglandular inflammation can lead to blurring of the gland margins (109, 110). There may be marked expansion along the lateral orbital wall and lateral rectus muscle (2, 109). Bone is usually spared (2); however, some cases may show bony destruction, though sclerosis or remodelling is more common (6). Contrast enhancement is common on both CT and MRI (**Figure 6**) (2, 3, 10, 12). On MRI, T1-weighted images may show the gland as hypointense-to-isointense compared to muscle, while T2-weighted images show the gland as hypointense-to-isointense compared to brain cortex. Lacrimal gland involvement is generally more T2-hypointense compared to most other lacrimal gland tumors (2, 9, 10).

In a study of 20 patients with idiopathic orbital inflammation, 85% exhibited an associated mass or infiltration, 75% had muscle cone involvement, 5% showed sinonasal involvement or bony changes, 30% had optic nerve involvement, and 15% had intracranial extension (107). As lacrimal gland imaging findings are non-specific, evaluation of other structures, such as the extraocular muscles, optic nerve, and orbital fat, may provide additional diagnostic insight.

#### 6.2.2 IgG4-ROD

IgG4-related disease is a multi-organ, immune-mediated disorder characterised by infiltration of IgG4-positive lymphoplasmacytic cells, leading to mass-like nodular enlargement of affected organs. When involving the orbit, it is termed IgG4-related ophthalmic disease (IgG4-ROD). Common manifestations include lacrimal gland enlargement and involvement of the extraocular muscles, orbital fat, infraorbital nerve, and other orbital structures (1, 111, 112).

**Figure 7:**
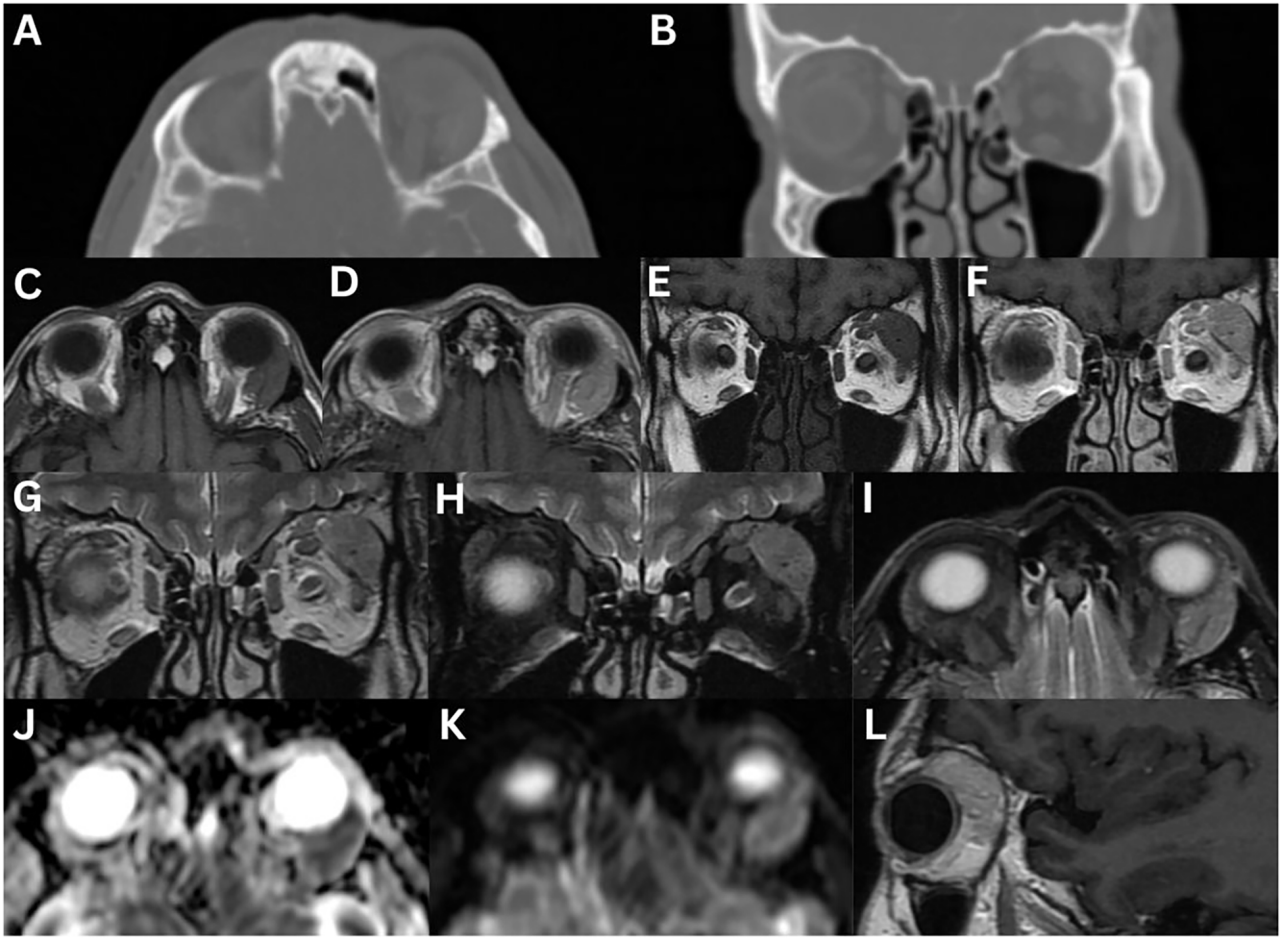
IgG4-related ophthalmic disease involving the left lacrimal gland. CT axial **(A)** and coronal **(B)** demonstrates no evidence of bony changes. MRI T1-weighted FLAIR axial **(C)** and coronal **(E)**, contrast-enhanced FLAIR axial **(D)** and coronal **(F)**, T2-weighted coronal **(G)**, fat-suppressed coronal **(H)** and axial **(I)**, and T1-weighted sagittal contrast-enhanced FLAIR **(L)** sequences demonstrate an isolated solitary enlargement of the left lacrimal gland. The lesion has a well-defined outline, with minimal vascularity increase in the affected portion of the lacrimal gland. Mild enhancement is observed following contrast administration. The gland is isointense on T1- and T2-weighted imaging relative to the adjacent extraocular muscles. There is evidence of significant restricted diffusion on axial ADC **(J)** and DWI **(K)**.

Imaging may demonstrate a diffuse mass with bilateral involvement (seen in approximately 84.8% of cases) (83), affecting both orbital and palpebral lobes (1, 104). The lacrimal glands typically maintain normal configuration and conform to the globe without indentation (85). Enlargement is often symmetrical, though asymmetry can occur (104, 111, 113). The condition may present with indistinct borders and ill-defined infiltration of orbital fat (104). Diffuse orbital involvement with infraorbital canal expansion and orbital nerve involvement favours a diagnosis of IgG4-ROD (104). Diffuse contrast enhancement is common, but not always present (1, 83, 104). Although bony involvement is rare (1), adjacent bone (not specific to the lacrimal gland), may show remodelling without destruction (**Figure 7**) (114). On MRI, the lacrimal gland is often low to intermediate intensity on T1-weighted sequences, and low intensity on T2-weighted sequences (104). The low T2 signal likely reflects intralesional increased cellularity and fibrosis (1, 114). Other sites of involvement include the optic nerve sheath, eyelid soft tissue, orbital fat, and the inferior optic nerve (83, 115).

In a case series of 33 patients with histologically confirmed IgG4-ROD of the lacrimal glands, the wedge sign was positive in 84.8% of cases. The lacrimal gland-orbital wall angle was acute in 15.2% of cases and obtuse in 84.8%. CT heterogeneity was observed in 14.3% of cases; on MRI, heterogeneity appeared in 36.4% of cases on T2-weighted imaging and 18.2% on contrast-enhanced T1-weighted imaging. Enhancement was high on CT in 96.4% of cases and high on MRI in 100%. T2-weighted signal intensity was isointense in 54.5% of cases and hypointense in 45.5%, relative to grey matter. Dynamic contrast-enhanced pattern revealed washout in 80% of cases, plateau in 0%, and prolonged enhancement in 20%. Extra-orbital involvement was noted in the infraorbital nerve (36.4%), paranasal sinus (72.7%), salivary gland (58.6%), nasopharynx (21.2%) and lymph nodes (54.5%) (83).

#### 6.2.3 Rosai-Dorfman-Destombes disease

Rosai-Dorfman-Destombes disease (sinus histiocytosis) is a non-neoplastic lymphoproliferative disorder marked by histiocytosis of the Rosai-Dorfman cell type (6). The lacrimal glands are affected in 8-25% of orbital cases (116). Radiologically, imaging characteristics vary (1), though the typical presentation is diffuse lacrimal gland enlargement, most often unilateral, with preservation of its normal shape and no bony involvement (1, 116). Bilateral cervical lymphadenopathy may be present (6), while isolated lacrimal gland disease without lymphadenopathy is rare. Orbital involvement can also precede systemic manifestations by several years (116).

Case reports describe proptosis and a well-defined lacrimal gland mass causing inferomedial globe displacement (117, 118). Although rare, focal lytic bone defects adjacent to the mass have been reported, which can mimic malignancy (1, 118). On MRI, the gland may exhibit intermediate signal intensity on both T1- and T2-weighted sequences, along with marked, homogeneous enhancement following contrast administration (117, 119). FDG-PET may demonstrate high FDG avidity in the affected gland (117).

The radiological findings of Rosai-Dorfman-Destombes disease are distinct from those of Langerhans cell histiocytosis, which presents as ill-defined, heterogeneous masses with bony lysis (1). The radiological findings are overall non-specific and patients are often misdiagnosed with conditions such as chronic dacryoadenitis or lymphoma (1).

#### 6.2.4 Sickle cell disease

Sickle cell disease is an autosomal recessive disorder caused by mutant haemoglobin, resulting in sickle-shaped red blood cells. Under hypoxic conditions, haemoglobin polymerises, leading to red blood cell sickling, vaso-occlusion, haemolysis, reperfusion injury, and microinfarctions (120–122). Lacrimal gland involvement, either unilateral or bilateral, has been reported, typically manifesting as lacrimal gland oedema. CT and MRI may show gland enlargement with homogeneous enhancement (2) and no bony changes (120). Volumetric analysis demonstrated a 32% increase in lacrimal gland size in sickle cell patients versus controls (p < 0.05) (122). Significant T2 shortening, both in peak and mean values, was observed in these patients compared to healthy controls, likely reflecting long-term processes such as iron accumulation or fibrosis secondary to vaso-occlusion and chronic inflammation, which is common in other organs affected by sickle cell disease. Similar T2 shortening has been noted in craniofacial bone marrow and salivary glands of sickle cell disease patients, as demonstrated through qMRI analyses (122).

### 6.3 Infectious

#### 6.3.1 Infectious dacryoadenitis (including abscess)

Acute dacryoadenitis is most commonly caused by infections, typically originating from the conjunctiva, skin, or via haematogenous spread in the setting of bacteraemia (1). Viral infections are more common than bacterial. It typically presents with unilateral, painful swelling of the superolateral orbit and proptosis (1, 2, 6). Imaging is generally unnecessary in typical, uncomplicated cases (1).

Imaging typically demonstrates unilateral, diffuse enlargement of both orbital and palpebral lobes, with preservation of the gland’s normal shape (1, 2, 10). There is marked contrast enhancement on both CT and MRI, particularly in the acute phase, reflecting hyperaemia, often with periglandular inflammation and fat stranding (1, 2, 10, 12). Inflammation may extend to adjacent muscles, causing superior and lateral rectus myositis (1, 2, 10). Bone involvement is uncommon (2). If scleritis is present, fluid may accumulate in Tenon’s space with uveoscleral enhancement (1, 10). Lymph node enlargement may also be observed (6). Assessment for orbital, cavernous sinus, or intracranial extension, including venous sinus thrombosis, is important (6).

Infectious dacryoadenitis can be complicated by abscess formation, for which MRI is more informative than CT (123). Abscesses are usually unilateral, presenting as glandular enlargement, with either indistinct or well-defined borders, and a central area of markedly lower density. Rim-enhancing lesions with thickened walls may also be present, along with displacement, flattening and indentation of the globe (124–128). On MRI, abscesses are hypointense on T1-weighted sequences and hyperintense on T2-weighted sequences, with DWI showing high signal and ADC maps showing low signal, indicating restricted diffusion (123, 124). DWI and ADC help differentiate abscesses from other cystic lesions (123). MRI may also reveal a cystic mass showing a fluid-fluid level, with the inferior fluid layer demonstrating increased signal intensity on DWI, indicating restricted water diffusion, which is consistent with an abscess (125).

#### 6.3.2 Tuberculosis

Tuberculosis, caused by *Mycobacterium tuberculosis*, is a multisystemic infectious disease (129). Ocular tuberculosis most frequently spreads haematogenously, primarily affecting the uveal tract because of its abundant blood supply. However, it can rarely involve extraocular structures, including the eyelid, conjunctivae, cornea, sclera, and lacrimal gland (129, 130). Imaging features of orbital tuberculosis include involvement of orbital bones and lacrimal gland, with soft tissue inflammation or abscess formation (129, 131, 132). Case reports of tuberculous dacryoadenitis may show variable heterogeneity and enhancement, be bilateral, and appear isodense to muscle on CT (130, 133). Proptosis, globe displacement, and regional lymphadenopathy may also occur (133).

### 6.4 Autoimmune

#### 6.4.1 Sarcoidosis

**Figure 8:**
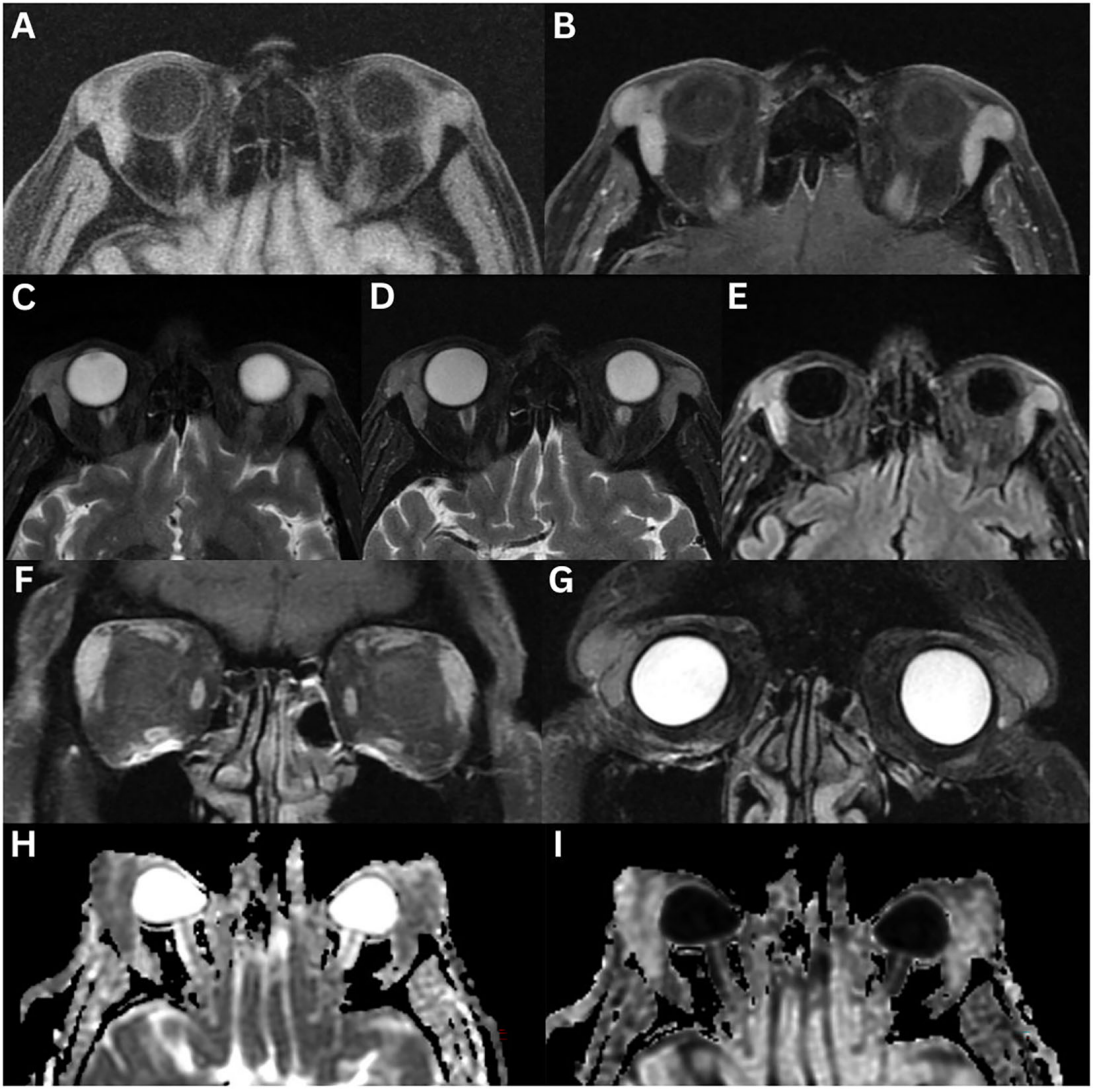
Sarcoidosis with bilateral lacrimal gland involvement. MRI T1-weighted axial fat-suppressed FLAIR **(A)**, axial fat-suppressed contrast-enhanced **(B)**, T2-weighted axial **(C)**, axial fat-suppressed **(D)**, axial fat-suppressed FLAIR **(E)**, T1-weighted coronal fat-suppressed contrast-enhanced **(F)**, and T2-weighted coronal fat-suppressed **(G)** sequences demonstrate asymmetric bilateral enlargement of the lacrimal glands, involving both orbital and palpebral lobes. The glands exhibit normal signal intensity on T1- and T2-weighted imaging, with normal homogeneous enhancement following contrast administration. Restricted diffusion is evident on axial ADC **(H)** and axial eADC **(I)**.

Sarcoidosis is a chronic, multisystem granulomatous inflammatory disorder that can affect almost any organ, most commonly the lungs, skin, and eyes (1). Lacrimal gland involvement occurs in 7-15% of patients and may represent the initial manifestation (1, 12). Histologically, it features noncaseating granulomas (6). Radiologically, lacrimal gland sarcoidosis typically presents as bilateral, symmetric diffuse enlargement of both orbital and palpebral lobes, without bony involvement. (**Figure 8**) (1, 2, 6, 12, 134). The glands typically show uniform contrast enhancement (1, 2, 12). On MRI, they demonstrate low-to-intermediate signal on T1-weighted sequences and high signal on T2-weighted sequences (2, 12).

Sarcoidosis can involve other orbital structures, leading to uveitis (anterior, posterior, or panuveitis), retinal vasculitis, optic nerve involvement, episcleritis, conjunctival granulomas, and keratoconjunctivitis (135). In a study of 13 patients with orbital sarcoidosis, associated findings included orbital masses or infiltration (54%), muscle cone involvement (15%), sinonasal involvement (7.7%), bony changes (7.7%), and optic nerve involvement (7.7%), with no intracranial extension (107). MRI may also reveal bilateral cervical lymphadenopathy, and enlargement of the parotid and submandibular glands (134). On Gallium-67 citrate imaging, symmetrical tracer uptake in the lacrimal and parotid glands, known as the ‘panda sign’, is highly suggestive of sarcoidosis, but has also been described in lymphoma following irradiation, Sjogren’s syndrome, and AIDS (6). Chest imaging is recommended to assess for mediastinal and hilar lymphadenopathy (1, 2, 12).

#### 6.4.2 Sjogren’s syndrome

Sjogren’s syndrome is a chronic autoimmune disorder primarily affecting the lacrimal and salivary glands, leading to sicca syndrome, characterised by dry eyes and dry mouth (1, 2, 12). Imaging findings of the lacrimal gland correlate with the temporal pathological changes, and may aid in assessing disease progression (1, 12). Early stages show bilateral, diffuse glandular enlargement due to lymphoplasmacytic infiltration, followed by progressive fatty deposition and eventual atrophy as fibrosis ensues (1, 2, 12). Glandular involvement therefore manifests as either enlargement or atrophy, without bony involvement (2).

**Figure 9:**
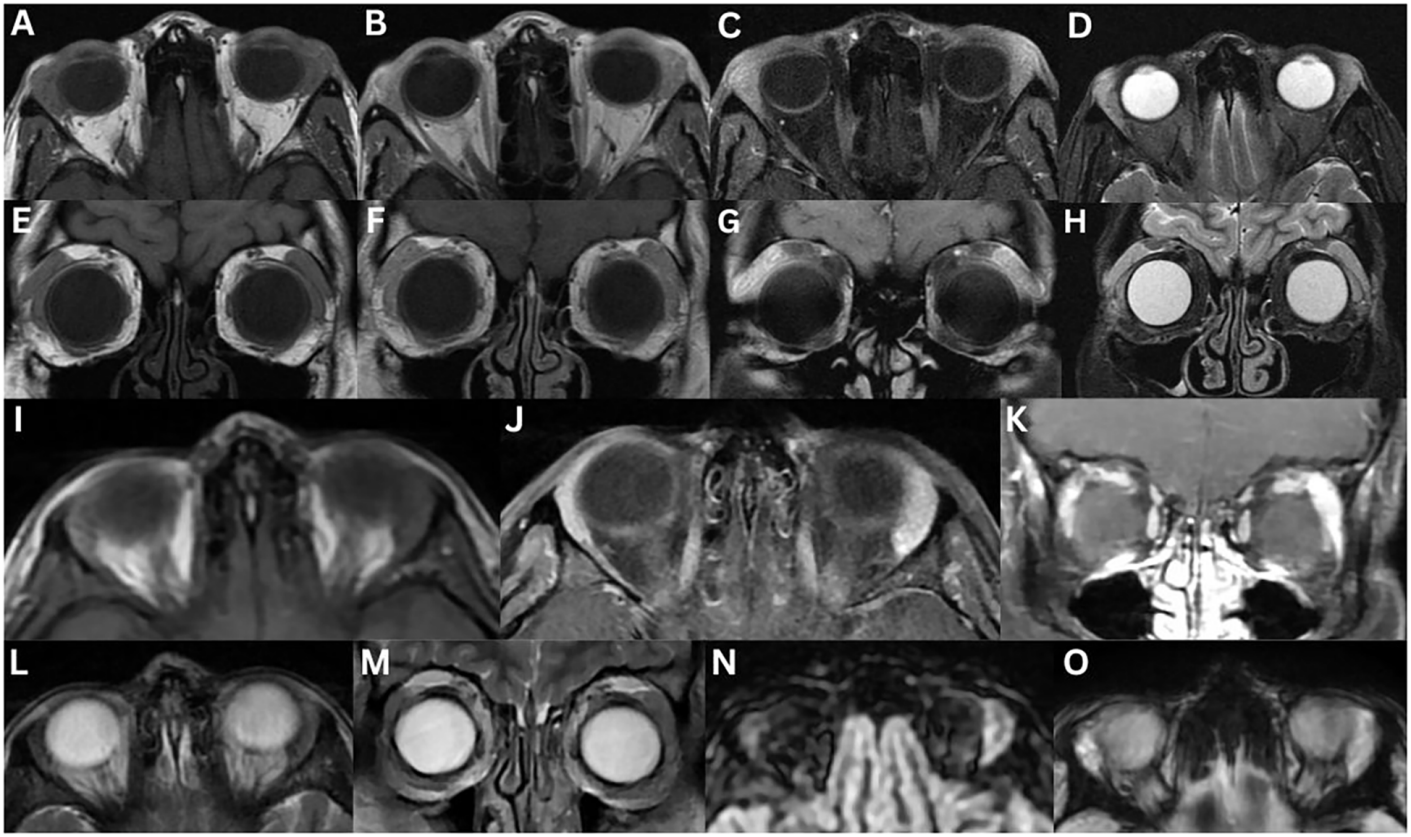
Sjogren’s syndrome with bilateral lacrimal gland disease **(A–H)**. MRI T1-weighted axial **(A)** and coronal **(E)**, T1-weighted contrast-enhanced axial **(B)** and coronal **(F)**, T1-weighted fat-suppressed contrast-enhanced axial **(C)** and coronal **(G)**, T2-weighted axial fat-suppressed **(D)**, and coronal STIR **(H)** demonstrates bilateral symmetrical enlargement of both lacrimal glands, including orbital and palpebral lobes with homogeneous signal intensity and homogeneous pattern of enhancement. In another patient with Sjogren’s syndrome **(I–O)**, MRI T1-weighted axial **(I)**, T1-weighted fat-suppressed contrast-enhanced axial **(J)** and coronal **(K)**, T2-weighted axial **(L)** and coronal propeller **(M)**, axial 3D DIR CUBE **(N)**, and axial 3D SWAN **(O)** sequences demonstrate asymmetrical enlargement of bilateral lacrimal glands. The enlargement of the left lacrimal gland affects the orbital lobe to a greater extent than the palpebral lobe. No focal mass is identified, and the gland demonstrates diffuse, homogeneous enhancement following contrast administration.

On MRI, the lacrimal gland is generally isointense to muscle on both T1- and T2-weighted imaging, but may demonstrate patchy high intensity areas on T1-weighted images due to fatty replacement (**Figure 9**) (1, 2, 12, 13). These high-intensity signals are suppressed on spectral presaturation with inversion recovery (SPIR) sequences, suggesting fat deposition (13). Glandular heterogeneity is reported in 48.3% to 100% of cases, with post-contrast images typically demonstrating heterogeneous enhancement (14). This finding on either US or MRI is considered suggestive of lacrimal gland involvement in Sjogren’s syndrome (14). DWI sequences have demonstrated significantly lower ADC values in affected glands compared to controls (6, 13, 14).

Ultrasound parameters measured in Sjogren’s syndrome include gland size, echotexture, shear-wave elastography (SWE) index, and colour doppler (14). While glandular heterogeneity increases diagnostic likelihood, gland size and the presence of fibrous bands or hyperechoic areas do not (14). SWE is a technique used to assess tissue stiffness. As glandular parenchyma atrophies and is replaced by fat, its mechanical properties change, which can be quantitatively measured using SWE. A cut-off value of 10.4 kPa is suggestive of Sjogren’s syndrome, demonstrating high specificity (97.6%) but lower sensitivity (70.6%), though requires further validation in larger cohorts (14). Colour doppler imaging shows an elevated resistive index and reduced diastolic flow in the lacrimal artery compared to controls (14, 15). Ultrasound can also identify echotextural changes indicative of fatty infiltration or atypical lymphocytic proliferation due to lymphoma, making it a valuable tool for monitoring morphological, volumetric, and structural changes over the course of disease progression, whether resulting from therapy, or possible degeneration of long-standing inflammation (15).

Patients with Sjogren’s syndrome have a 16-18-fold increased risk of B-cell lymphoma compared to the general population. Thus, asymmetrical glandular enlargement, particularly with a distinct enhancing intraglandular mass, with or without bony scalloping, should raise suspicion for malignancy. Furthermore, associated lymphadenopathy or splenomegaly further support this diagnosis (1).

#### 6.4.3 Thyroid eye disease

**Figure 10:**
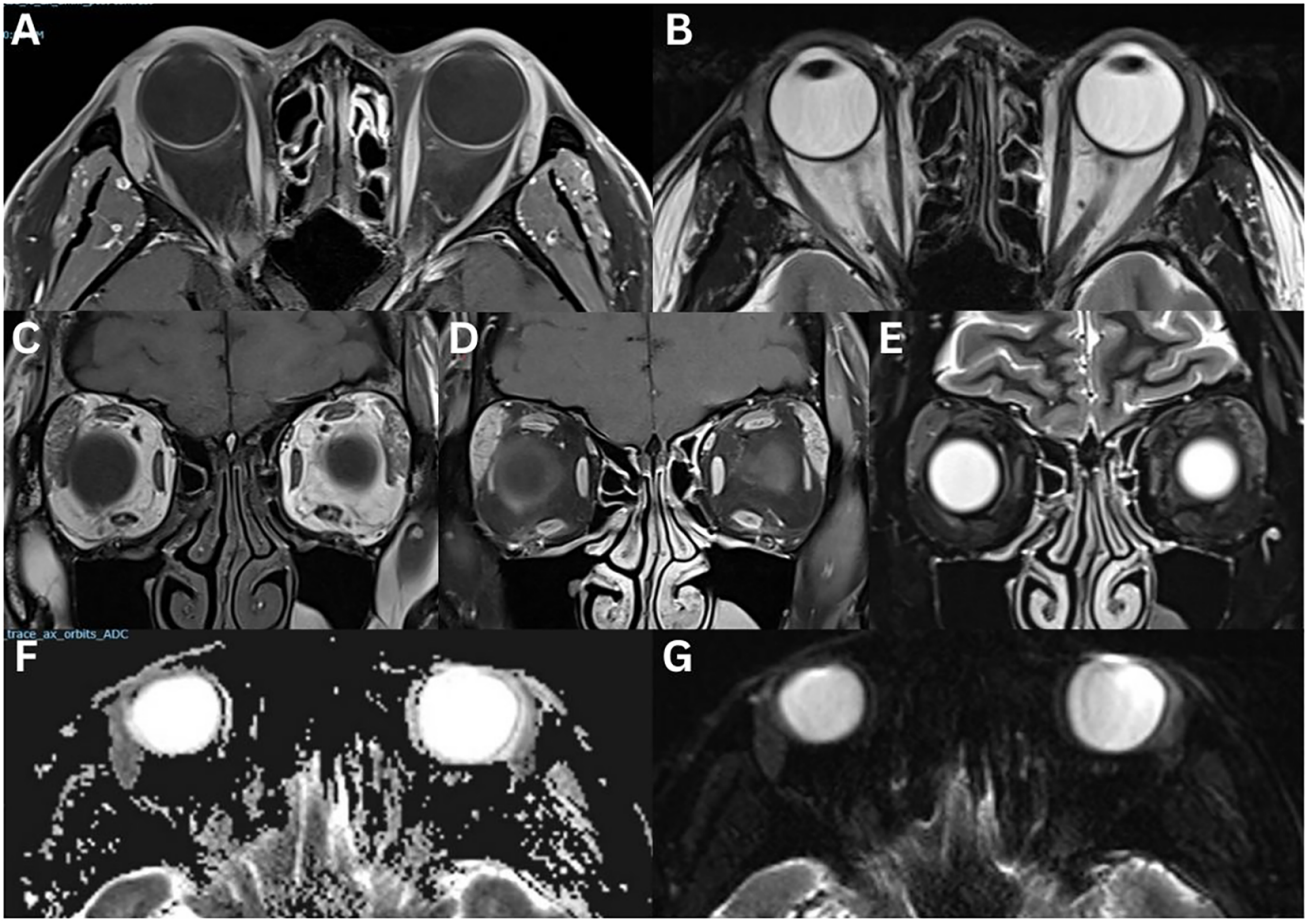
Thyroid eye disease with asymmetrical bilateral lacrimal gland involvement. MRI T1-weighted axial fat-suppressed contrast-enhanced **(A)**, T2-weighted axial **(B)**, T1-weighted coronal **(C)**, coronal fat-suppressed contrast-enhanced **(D)**, and T2-weighted coronal **(E)** sequences demonstrate bilateral enlargement of the lacrimal glands, with moderate enhancement following contrast administration. The glands appear mildly hyperintense on T2-weighted imaging, relative to the adjacent extraocular muscles. There is a restricted diffusion pattern on axial ADC **(F)** and axial TRACE **(G)**.

Thyroid eye disease (TED) is an autoimmune inflammatory disorder of the orbit, occurring in 25-80% of patients with Graves’ disease. While lacrimal gland enlargement is a common feature of TED and may serve as an initial presenting sign, it is seldom observed in isolation; most patients present with concurrent radiological signs, such as lacrimal gland prolapse, proptosis, extraocular muscle enlargement, and orbital fat involvement (**Figure 10**) (136, 137). Enlargement, assessable by volume, area, and length measurements, is typically bilateral and symmetrical (138).

Compared with controls, patients with TED demonstrate increased maximum axial and coronal area, axial length and width, coronal width, and DTI-ADC/MD values (137). An ADC cut-off value of 1.62 x 10^–3^ mm²/s demonstrates high sensitivity (97%) and specificity (95%) for differentiating TED from controls (139).

Active TED is associated with significantly larger lacrimal gland herniation, maximum coronal area, and higher DWI-ADC values than inactive disease (137). The signal intensity ratio of the lacrimal gland to the ipsilateral temporal muscle on fat-suppressed T2-weighted imaging is also higher in active disease (140). An ADC threshold of 1.76 x 10^–3^ mm²/s differentiates active from inactive TED, with 65% sensitivity and 100% specificity (139).

On MRI, the lacrimal gland appears hyperintense on T2-weighted imaging relative to the ipsilateral muscle, likely reflecting inflammatory oedema (140, 141).

#### 6.4.4 Granulomatosis with polyangiitis

Granulomatosis with polyangiitis is one of the more prevalent ANCA-associated vasculitides, primarily affecting the upper and lower respiratory tracts and kidneys (107). However, any organ may be affected, including the orbit, which is involved in 40–50% of cases (6, 12, 107). Lacrimal gland disease may occasionally serve as the initial sign, preceding other orbital or systemic manifestations (12, 107).

**Figure 11:**
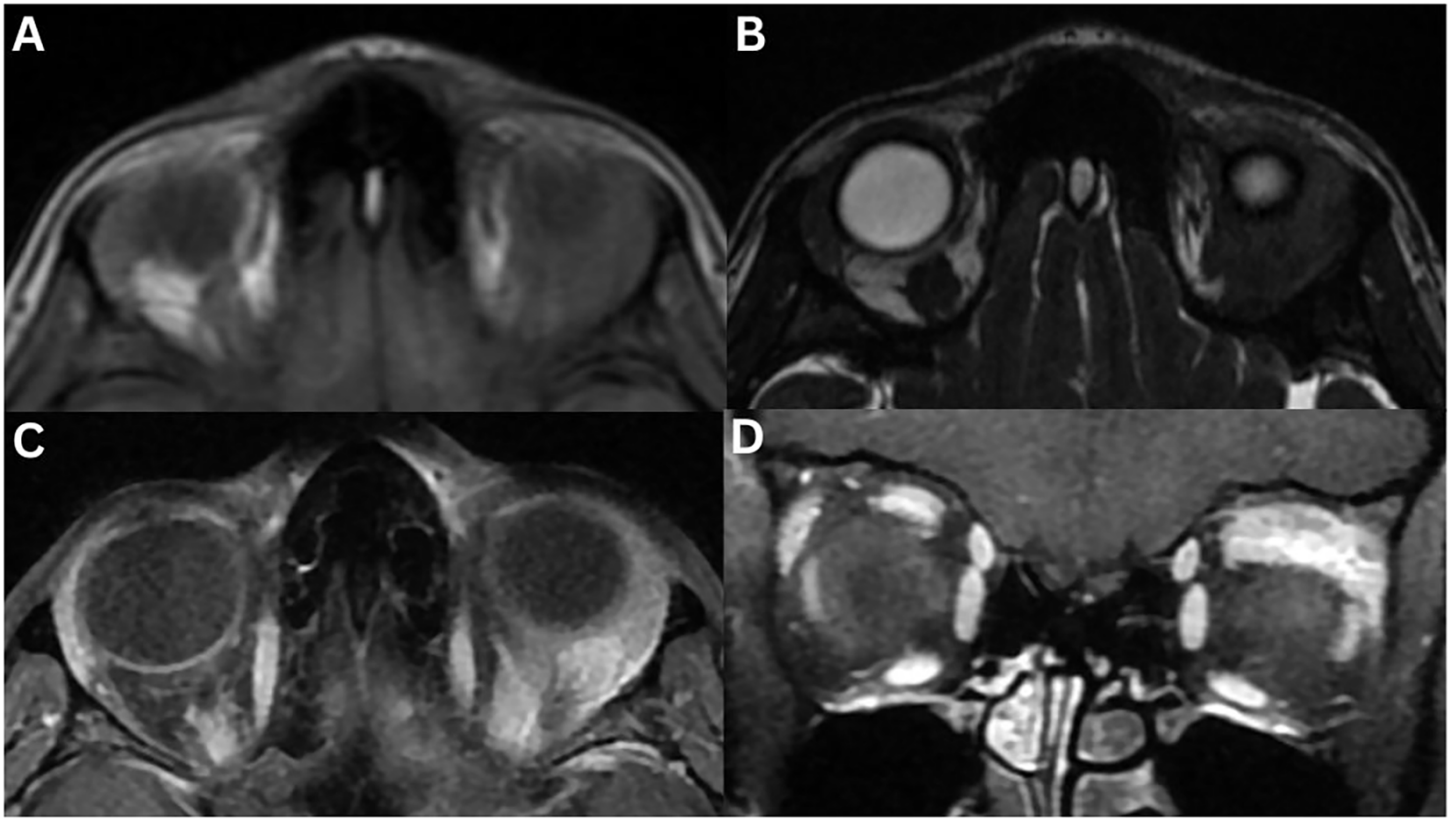
Granulomatosis with polyangiitis involving the left lacrimal gland. MRI T1-weighted axial **(A)**, axial 3D Fiesta-C **(B)**, T1-weighted fat-suppressed contrast-enhanced axial **(C)** and coronal **(D)** demonstrate enlargement of the lacrimal gland including the orbital and palpebral lobe. The gland is homogeneous with well-defined margins. It is isointense on T1-weighted imaging and hyperintense on T2-weighted imaging, relative to the extraocular muscles. There is significant enhancement following contrast administration.

Lacrimal gland involvement may be unilateral or bilateral (107, 115, 142). The gland may appear diffusely enlarged with well-defined margins (143, 144). On CT, osseous erosion and globe indentation may be present, occasionally linked with necrotising scleritis (9, 85, 107, 143). The lesion is typically homogeneous and of similar attenuation to muscle (12, 115). On MRI, T1-weighted imaging may show a hypointense mass relative to the extraocular muscles, along with soft tissue infiltration of orbital fat. On T2-weighted imaging, the mass often demonstrates variable heterogeneity, but is typically hyperintense compared to muscle (**Figure 11**) (9, 12, 115). There may be normal diffusion on DWI. Contrast-enhanced CT and MRI may reveal moderate, variably heterogeneous enhancement (12, 115, 143, 144). FDG-PET CT imaging may reveal increased FDG avidity in the affected gland (142).

Associated findings may include sinonasal disease, including septal perforation, as well as thickening of the optic nerve sheath (9, 12, 115). In a study of seven patients with lacrimal gland involvement, 57% had an associated mass or infiltration, 29% had muscle cone involvement, 29% had bony changes, 43% had sinonasal involvement, 14% had optic nerve involvement, and none had intracranial extension (107). Early sinonasal disease often presented as inflammatory mucosal changes and subtotal opacification of the ethmoid and maxillary sinuses, which were non-contiguous with the orbital disease (107). In one case, progressive nasal septal destruction progressed to extensive sinonasal disease and collapse of the nasal bridge (107).

#### 6.4.5 Eosinophilic granulomatosis with polyangiitis (EGPA)

**Figure 12:**
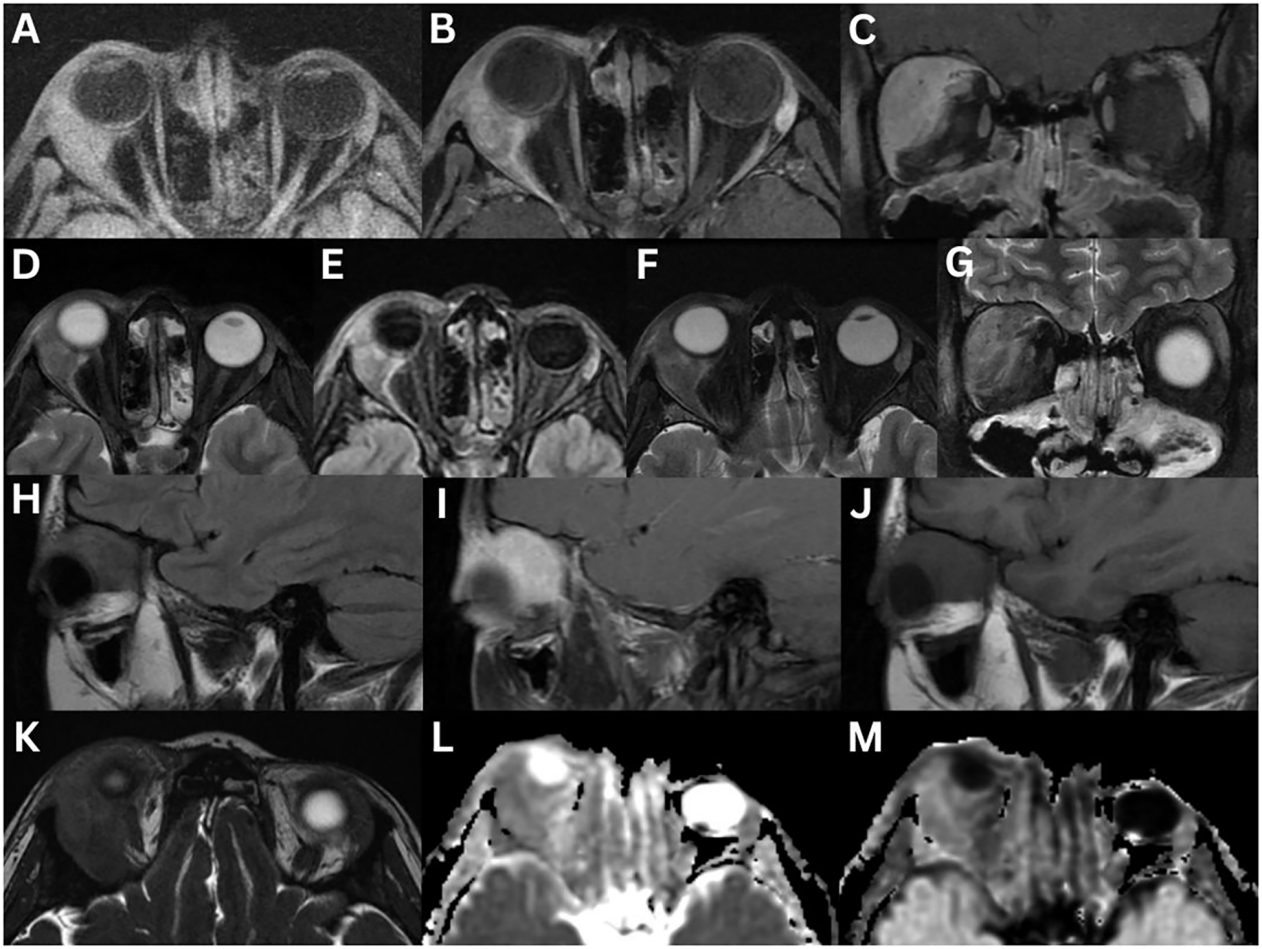
Eosinophilic Granulomatosis with Polyangiitis with involvement of the right lacrimal gland. MRI T1-weighted axial fat-suppressed FLAIR **(A)** and fat-suppressed contrast-enhanced axial **(B)** and coronal **(C)**, T2-weighted axial **(D)**, axial fat-suppressed FLAIR **(E)**, axial fat-suppressed **(F)** and coronal fat-suppressed **(G)**, T1-weighted sagittal FLAIR **(H)**, and sagittal fat-suppressed contrast-enhanced **(I)**, and T2-weighted sagittal FLAIR **(J)** sequences demonstrate an enlarged right lacrimal gland with an ill-defined outline and heterogeneous contrast enhancement. The lesion encases the adjacent superior rectus complex and right lateral rectus muscle, as well as the adjacent orbit displacing them inferomedially. Axial 3D Fiesta is also shown **(K)**. There is mild diffusion restriction on axial ADC **(L)** and axial eADC **(M)**.

EGPA is a rare systemic vasculitis affecting small and medium-sized vessels. Although orbital involvement is rare, lacrimal gland involvement has been documented (104, 145). However, isolated lacrimal gland involvement is exceedingly rare (104). In a series of 45 patients with orbital disease, 4.4% had lacrimal gland involvement, either unilateral or bilateral (104). MRI may show an enlarged lacrimal gland with ill-defined borders and heterogeneous contrast enhancement (**Figure 12**), sometimes with inferomedial displacement and intraconal extension with soft tissue stranding. Bony involvement is uncommon (145–147). Beyond the lacrimal gland, EGPA typically presents with diffuse orbital inflammation, including enlargement and enhancement of extraocular muscles, peribulbar tissues, optic nerve, and orbital fat (104, 145, 147, 148).

#### 6.4.6 Crohn’s disease

Ocular involvement occurs in 3.5 - 11.8% of inflammatory bowel disease cases, including Crohn’s disease and ulcerative colitis (149). Dacryoadenitis is a rare complication, more commonly seen in Crohn’s disease, typically affecting both lacrimal glands (149). Bony involvement is rare (150–154). The gland may appear oblong, distinguishing it from the rounded shape of epithelial tumours, and may extend beyond the orbital rim, consistent with palpebral lobe involvement (153). On MRI, the gland may demonstrate intermediate signal on both T1- and T2-weighted imaging, with contrast enhancement (153, 155). One case reported lacrimal gland inflammation as the initial presentation of ulcerative colitis, with CT showing bilateral involvement without bony changes (149).

## 7 Structural abnormalities

### 7.1 Developmental orbital cysts (involving the lacrimal fossa)

Developmental orbital cysts are congenital lesions that include dermoid cysts, epidermoid cysts, and teratomas (1). Dermoid cysts are the most common, accounting for 3-9% of all orbital tumours, arising from ectodermal sequestration, typically in the superior temporal quadrant near the zygomaticofrontal suture (1, 6, 9, 10, 12, 19). Although not true lacrimal gland tumours, they arise from epithelial rests within the lacrimal fossa (12, 19). Most grow slowly, though some may exhibit rapid growth in adults (10).

On CT, dermoid cysts appear as low-density, fat-like lesions, often with rim calcifications and smoothly outlined osseous changes, including scalloping and localised excavation. They generally show no contrast enhancement (1, 6, 9, 10, 19). MRI typically demonstrates low signal intensity on T1-weighted imaging with minimal or no contrast enhancement, and high signal intensity on T2-weighted imaging, often exhibiting a characteristic fat signal intensity (10, 12, 19). In rare cases, cyst rupture can cause an inflammatory reaction with irregular thickened margins and associated orbital inflammation (6, 19). Dermoid cysts usually remain confined to suture lines, such as the frontozygomatic suture line, whereas lacrimal gland lesions extend beyond it (6). If the tumour extends through the suture line, it may appear on both sides of the bone, known as a “dumbbell” dermoid (12, 19). Epidermoid cysts may have similar imaging features but do not typically demonstrate calcification (10, 12). Like dermoid and epidermoid cysts, teratomas typically present as hypodense lesions on CT with characteristic fat densities (1).

### 7.2 Lacrimal ductal cysts (dacryops)

Lacrimal ductal cysts, or dacryops, account for 3-6% of all lacrimal gland lesions (20, 156), arising primarily in the palpebral lobe where excretory ducts emerge onto the conjunctiva (6, 19, 20). These slow-growing cysts may occasionally be bilateral (6, 157) and result from obstruction of the lacrimal gland ducts (6, 19). While often idiopathic, they may also be caused by blockage from a small, slow-growing lacrimal tumour, a consideration radiologists should be mindful of (6). Additionally, malignant transformation of the cyst’s epithelial wall into squamous cell carcinoma has been reported (6).

**Figure 13:**
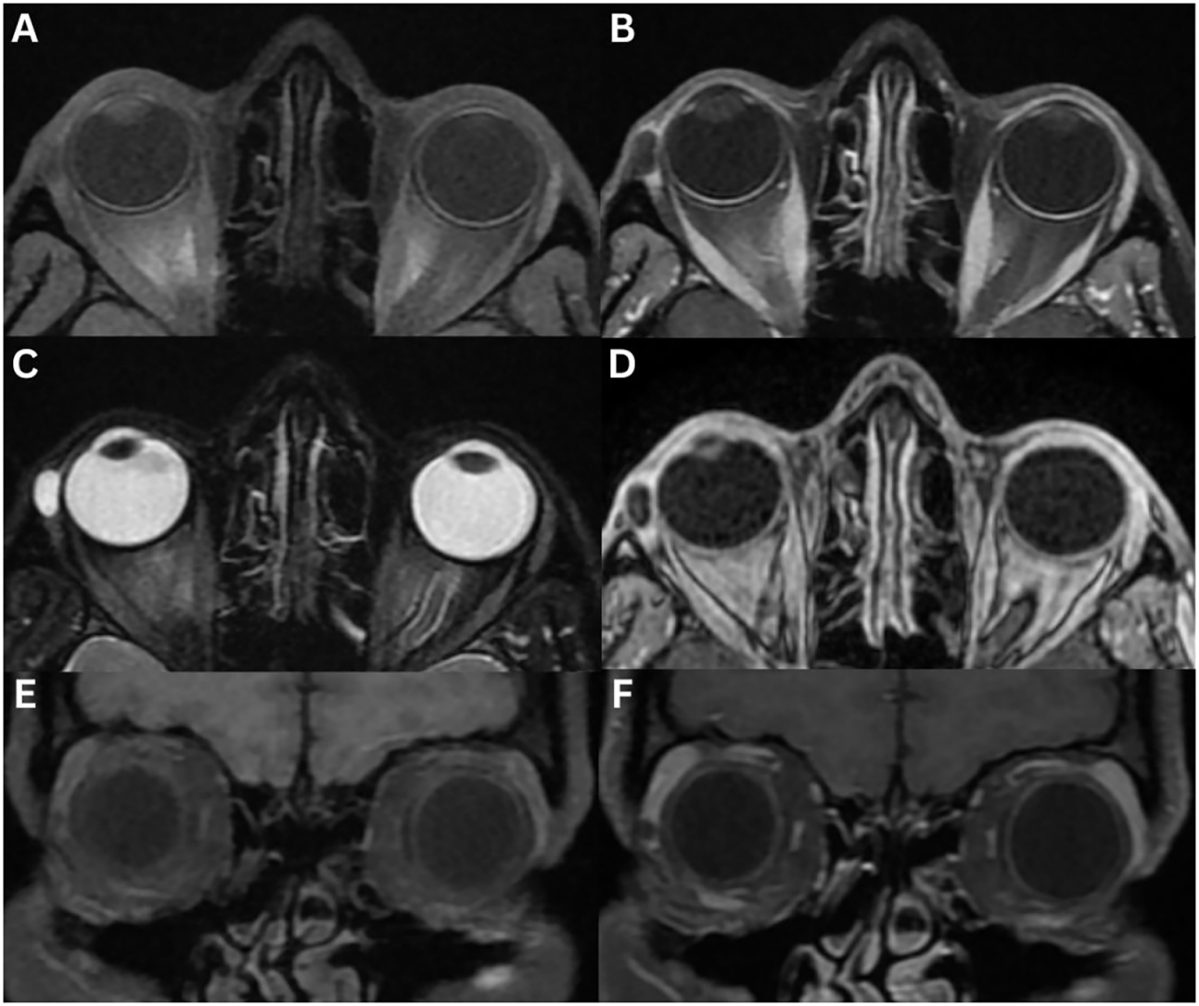
Dacryops of the right lacrimal gland. MRI T1-weighted axial fat-saturated **(A)** and axial fat-saturated contrast-enhanced **(B)**, T2-weighted axial fat-suppressed **(C)**, axial 3D contrast-enhanced **(D)**, and T1-weighted coronal fat-suppressed **(E)** and coronal fat-suppressed contrast-enhanced **(F)** sequences demonstrate a right superficial palpebral lobe cystic lesion. There is no contrast enhancement and no mass effect on adjacent structures.

Imaging helps localise deep orbital dacryops, assess bony extension, and differentiate them from other cystic lesions like dermoid cysts, frontal mucoceles, aneurysmal bone cysts, and implantation cysts (6, 156, 158). Radiologically, they appear as well-defined cystic lesions filled with clear fluid, clearly demonstrated on US, CT, and MRI (**Figure 13**) (6, 19, 20). Typically, there is no nodular or thick enhancement, and no bony involvement. Palpebral lobe cysts lie anterior to the orbital rim (19, 156, 158). Small intralesional calcifications may occasionally be present. Typically, there is no contrast enhancement; however, soft tissue density variants can demonstrate variable enhancement (6, 156). On US, they appear as thin-walled, spherical cysts with low internal reflectivity and a hyperechoic wall (156, 158). Nuclear medicine scans, though rarely employed, have demonstrated high uptake of Tc99m-pertechnetate (156).

### 7.3 Amyloidosis

Amyloidosis refers to a group of disorders characterised by extracellular deposition of insoluble protein fibrils, which can be localised or systemic. Lacrimal gland involvement is rare (9, 159, 160), and radiological findings are infrequently reported. Case reports describe CT findings of well-defined, homogeneously enlarged lacrimal glands, occasionally containing intralesional calcifications. The mass may mould around the globe without causing bony erosion or destruction (159–161). It often appears isodense to muscle and may enhance with contrast (159). On MRI, the lesion may show high signal intensity on T1-weighted imaging and low signal intensity on T2-weighted imaging, often with rim enhancement (159, 161). Extraocular muscle enlargement and surrounding soft tissue infiltration may also be evident (9, 159, 160).

### 7.4 Lipoma

A lipoma is a benign soft-tissue tumour composed of mature adipocytes enclosed within a thin fibrous capsule, typically exhibiting slow, progressive growth (162). While orbital lipomas are rare, cases involving the eyelid have been reported, and lacrimal gland involvement is exceptionally uncommon (162). It remains unclear whether these tumours arise within or adjacent to the gland. On MRI, a simple lipoma appears as a homogeneous fatty mass with minimal or no septa and little to no post-contrast enhancement. CT reveals attenuation consistent with fat (10). In one case, congenital absence of the lacrimal gland was associated with a lipoma occupying its location; orbital MRI demonstrated a lesion with signal intensity identical to subcutaneous fat across all sequences, with suppression on fat-saturation techniques and no contrast enhancement (163).

## 8 Vascular anomalies

Vascular anomalies are broadly classified into two categories: vascular tumours and vascular malformations. Vascular tumours are characterised by proliferative changes of endothelial cells, whereas vascular malformations are structural abnormalities of the vasculature without endothelial proliferation. Vascular tumours can be further subdivided into three groups: benign, locally aggressive or borderline, and malignant (164). Vascular malformations can be further subclassified based on the type of blood vessel and anomaly, including capillary, lymphatic, venous, and arteriovenous malformations (164). When the lacrimal gland is involved, it remains unclear whether the vascular anomaly originates within the gland itself or simply abuts and displaces it.

### 8.1 Vascular tumours (capillary haemangiomas and epithelioid haemangioendothelioma)

Capillary haemangiomas are benign vascular tumours of endothelial cells, and are the most common orbital tumour in infancy. They typically present within the eyelid, anterior to the globe (9). On CT, they may appear as a well-defined, homogeneous mass without bone involvement (165). In a case involving the lacrimal sac and duct, MRI revealed a mass with low intensity on T1-weighted imaging and intermediate to high intensity on T2-weighted imaging, with contrast-enhancing intratumoural vessels (9). The lacrimal gland may be displaced (1).

Epithelioid haemangioendothelioma is a rare vascular tumour with clinical and histopathological features intermediate between haemangioma and angiosarcoma (166). One reported case highlighted lacrimal gland involvement, with MRI revealing a homogeneous, low intensity mass in the palpebral lobe on T1-weighted imaging, appearing hypointense relative to normal gland tissue. On T2-weighted imaging, the lesion was heterogeneous with high signal intensity and demonstrated diffuse, bright post-contrast enhancement (166).

### 8.2 Vascular malformations (cavernous haemangiomas and venolymphatic and lymphatic malformations)

Cavernous haemangiomas, or orbital cavernous venous malformations, are common benign orbital masses in adults, commonly located in the lateral intraconal space (1). Considered a congenital malformation, they enlarge slowly over time, often due to haemodynamic changes (1). These changes lead to the development of new vascular spaces, gradually increasing the size of the lesion (167). Imaging may show a well-circumscribed, enhancing, homogeneous solid mass, with progressive growth and displacement of surrounding structures (1, 167–169). Bony remodelling may occur, but calcification, bony invasion, or significant periglandular infiltrative changes are generally absent (167, 169, 170). On MRI, the lesion typically appears hypointense on T1-weighted images and hyperintense on T2-weighted images, with visible flow voids (1, 168, 169). Punctate foci of low T2 intensity may reflect blood degradation products or small phleboliths (169). Post-contrast, the lesion enhances heterogeneously (168, 169).

Venolymphatic and lymphatic malformations are hamartomatous, slow-flow vascular malformations containing lymphatic and venous components. They grow slowly and may encapsulate or infiltrate surrounding structures. On CT and MRI, they typically appear poorly defined, lobulated, and trans-compartmental, often exhibiting fluid-fluid levels (1). On CT, a case of adult orbital lymphatic malformation involving the lacrimal gland showed a well-defined, heterogeneous mass with scattered calcifications and contrast enhancement, without orbital or bony invasion (171).

## 9 Histiocytic disorders

### 9.1 Langerhans cell histiocytosis

Langerhans cell histiocytosis (LCH) is a rare multisystem disease characterised by clonal proliferation of Langerhans cells. Orbital involvement is uncommon and typically confined to the bony orbit, with secondary involvement of orbital soft tissues (1, 6, 172, 173). Bone is the most common site, while isolated lacrimal gland involvement is exceedingly rare. When present, it usually occurs with a focal lytic bone lesion, often at the superior orbital rim, accompanied by a soft tissue mass (1, 172). Unlike primary lacrimal gland malignancies, bony destruction is not contiguous with lacrimal gland swelling (1).

**Figure 14:**
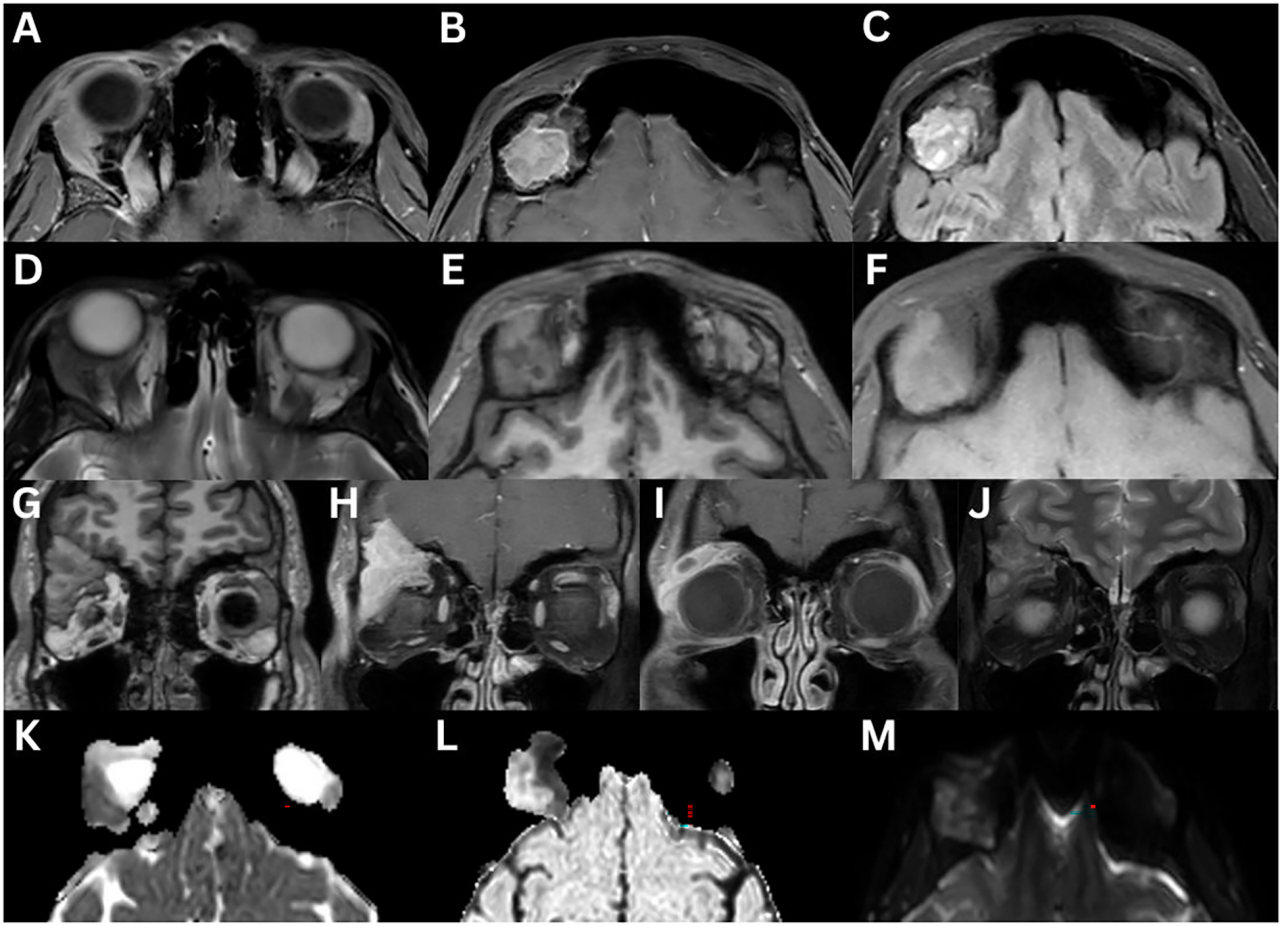
Langerhans cell histiocytosis with involvement of the right lacrimal gland. MRI axial T1-weighted fat-suppressed and contrast-enhanced **(A, B)**, axial contrast-enhanced FLAIR SPIR **(C)**, T2-weighted axial **(D)**, T1-weighted axial **(E)**, axial fat-suppressed **(F)**, coronal **(G)** and coronal contrast-enhanced DIXON **(H, I)**, and T2-weighted coronal DIXON **(J)** sequences demonstrate a heterogeneous mass with heterogeneous enhancement following contrast administration. The mass is isointense on T1-weighted imaging and mildly hyperintense on T2-weighted imaging, relative to adjacent extraocular muscles. There is restricted diffusion on axial ADC **(K)**, axial eADC **(L)** and axial DWI SENSE **(M)**.

Imaging shows bilateral, symmetrical enlargement of the lacrimal glands, involving both orbital and palpebral lobes (1). On MRI, lesions demonstrate a heterogeneous signal on T1-weighted imaging and are heterogeneously hyperintense on T2-weighted imaging, relative to muscle (**Figure 14**). The heterogeneity is likely due to varying levels of lipid deposition and haemorrhage (1). One case showed exophthalmos with osteolytic lesions with sclerotic delineation in the frontal, zygomatic, and maxillary bones, without extension into the adjacent orbital soft tissues. There was also bilateral optic nerve thickening, contrast enhancement, and a T2-hyperintense signal surrounding the optic nerve sheath (173).

### 9.2 Adult orbital xanthogranulomatous disease

Adult orbital xanthogranulomatous disease (AOXGD) is a rare granulomatous orbital disorder characterised by local or systemic proliferation of non-Langerhans cell histiocytes (174). It encompasses a heterogeneous group of rare, idiopathic inflammatory conditions, classified into four distinct syndromes: adult-onset xanthogranuloma (AOX), adult-onset asthma and periocular xanthogranuloma (AAPOX), necrobiotic xanthogranuloma (NBX) and Erdheim-Chester disease (ECD) (174). Lacrimal gland involvement is uncommon, typically unilateral, though bilateral cases occur (6, 10).

Imaging may demonstrate mild lacrimal gland enlargement, proptosis, extraocular muscle involvement, increased orbital fat, and an infiltrative soft tissue mass extending posteriorly around the globe (10, 175). Encapsulation of the optic nerve, bone destruction, and intracranial extension are less common (176). AAPOX is rare and may also involve the lacrimal gland, alongside other radiological findings (176). Orbital involvement in ECD is frequently multicompartmental, presenting with bilateral, symmetrical intraconal and extraconal soft tissue involvement, including the lacrimal glands (1, 177, 178). Extraocular muscles may also be affected (179). Additionally, diffuse bony sclerosis is commonly observed (1). While bony destruction is not generally observed in AOXGD, it has been reported in some ECD cases (174). The mass effect of the lesion can lead to optic nerve tortuosity (1, 179). MRI may demonstrate heterogeneously enhancing lacrimal glands (177).

## 10 Superolateral orbital lesions

Lesions in the superolateral orbit may be misinterpreted for primary lacrimal gland pathology. They can abut or invade the lacrimal gland, cause prolapse, or induce peritumoral inflammation resulting in dacryoadenitis. In addition, gland prolapse itself may mimic displacement by a mass (180).

Conjunctival squamous cell carcinoma, usually in the interpalpebral portion or limbus, can infrequently extend into the orbit and mimic a lacrimal gland mass (10). On MRI, these tumours exhibit low signal intensity on T1-weighted images and moderate-to-high signal intensity on T2-weighted images, with homogeneous contrast enhancement (181). Areas of intratumoural necrosis, when present, result in a heterogeneous signal with non-enhancing regions (181). Features may include superficial ulceration, subcutaneous protrusion, ill-defined margins, and peritumoural fat stranding; the latter reported in 93% of cutaneous tumours with height of >4mm (181). Rarely, direct spread into the lacrimal gland has been reported. In one case, MRI findings showed diffuse gland enlargement and enhancement without adjacent muscle or bone involvement (182). In two other cases, contrast enhancement from the tumour extended into the lacrimal gland, suggesting infiltration. However, histopathological examination revealed dacryoadenitis without evidence of squamous cell carcinoma infiltration, highlighting peritumoral inflammation as the cause of dacryoadenitis (181).

Meibomian gland carcinoma, a rare tumour accounting for less than 1% of all eyelid tumours, may secondarily invade the orbit, presenting as a solid nodule in the lacrimal gland fossa. This can also mimic a primary lacrimal gland tumour (183).

The palpebral lobe of the lacrimal gland may prolapse into the subconjunctival space of the superolateral fornix. This may result from glandular enlargement, prolapse of the orbital lobe, or occult herniation of orbital fat (184). This prolapse can be mistaken for a mass or interpreted as a mass causing the prolapse.

Subconjunctival fat prolapse, which may lead to lacrimal gland prolapse in some cases (184, 185), or mimic lacrimal gland pathology, is an acquired lesion characterised by the herniation of intraconal fat due to weakness of the Tenon capsule, often resulting from ageing, trauma, or surgery (185). Imaging may reveal bilateral involvement with fat continuous with the intraconal fat, extending anteriorly between the lateral wall of the globe medially and the lateral rectus muscle and lacrimal gland laterally. The herniated fat appears homogeneous, with CT attenuation and T1- and T2-weighted MRI signal intensities identical to those of intraorbital fat. There was no calcifications or significant post-contrast enhancement (186).

Dermolipoma, a congenital dermis-like connective and adipose tissue tumour (186), typically appears as a unilateral, crescent-shaped fatty mass at the temporal or superotemporal epibulbar region, lateral to the globe, anterior to the lateral rectus muscle insertion, and medial to the lacrimal gland. It is not connected to the intraconal fat and appears as a homogeneous fatty mass, with CT and MRI signal intensities identical to those of intraorbital fat. Similarly, there is no associated calcification or enhancement post-contrast (186).

**Figure 15:**
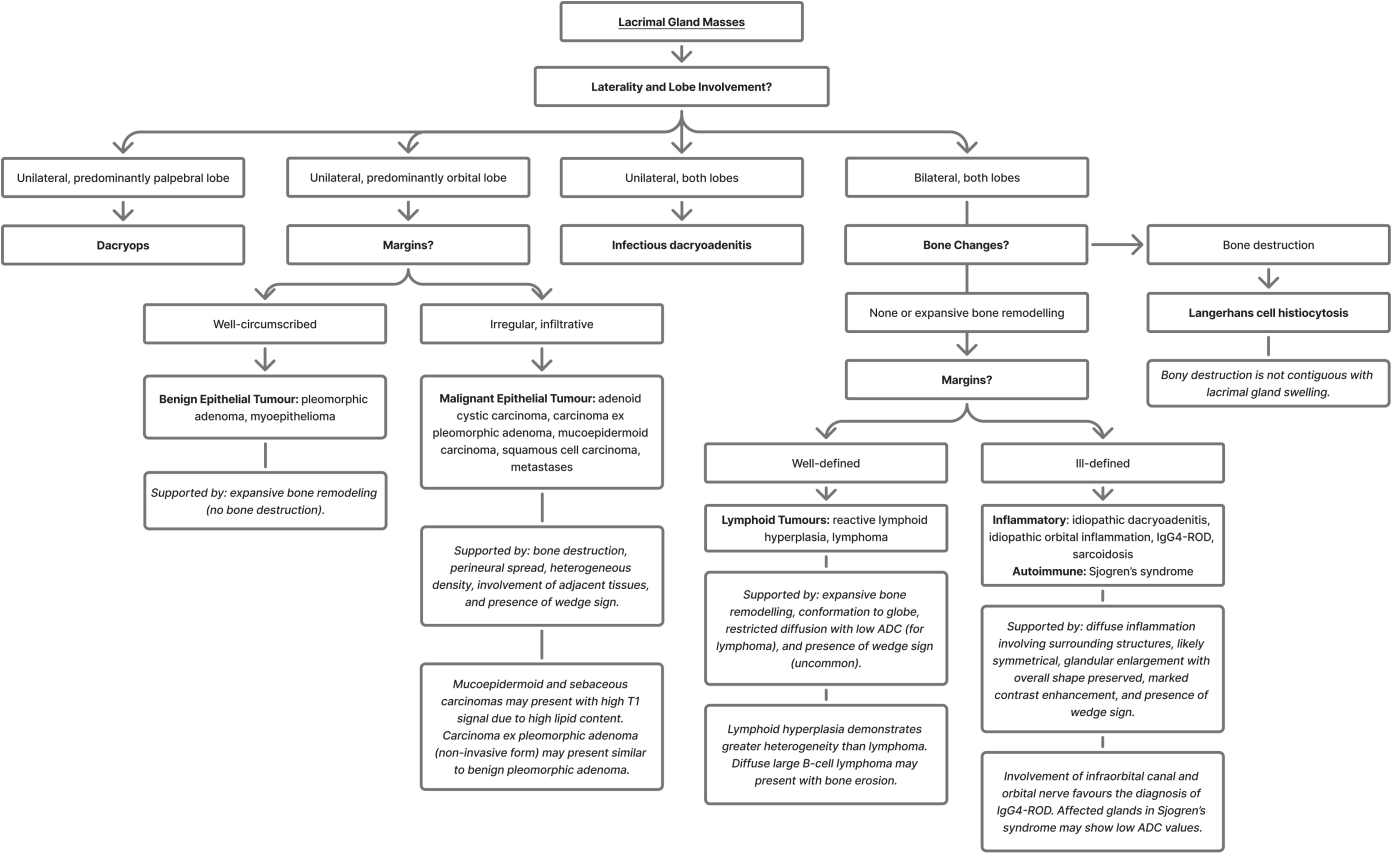
Pattern-based radiological decision-making flowchart for lacrimal gland lesions.

A concise summary of the pattern-based radiological approach and its key differential diagnoses is provided in **Figure 15** and **Table 1**.

**Table 1:** Key imaging features for lacrimal gland lesions.

Feature	Benign epithelial tumours	Malignant epithelial tumours	Lymphoid tumours	Inflammatory conditions
Laterality	Usually unilateral	Usually unilateral	Often bilateral	Often bilateral
Lobe Involvement	Predominantly orbital lobe; palpebral lobe in up to 10%	Predominantly orbital lobe	Diffuse involvement of both lobes	Diffuse involvement of both lobes
Margins	Well defined, regular, smooth	Irregular, ill-defined, infiltrative	Well-defined	Ill-defined, blurred
Shape	Round, oval	Lobulated, nodular, globular	Oblong, rounded; moulds around globe with concave inner margins	Despite enlargement, overall shape of gland is preserved
Growth Pattern	Slow, expansile	Rapid, aggressive	Indolent; moulding rather than displacement	Variable; typically subacute
Globe Displacement	Inferonasal displacement common	Variable; often more pronounced	Moulding with minimal displacement	Minimal or no displacement
Bone Changes	Remodelling, scalloping; destructive changes rare	Erosion, invasion, or destruction; sclerosis possible	Remodelling; erosion uncommon	Rare; remodelling may occur, destruction rare
T1 MRI Signal	Hypo- to isointense	Variable; typically hypo- to isointense, though may be hyperintense in lipid-rich tumours (e.g. mucoepidermoid, sebaceous carcinoma)	Isointense	Isointense
T2 MRI Signal	Iso- to hyperintense	Variable	Hypo- to isointense	Hypo- to isointense
Contrast Enhancement	Moderate, often homogeneous	Moderate-high, typically heterogeneous	Moderate-high; lymphoid hyperplasia is more heterogeneous than lymphoma	Homogeneous, intense
Diffusion/ADC	No diffusion restriction, high ADC	Variable; often shows diffusion restriction with low ADC in high-cellularity tumours	Marked diffusion restriction; low ADC	No diffusion restriction, high ADC
Wedge Sign	Absent	Often present; highly suggestive of malignancy	Common	Common
Perineural Spread	Absent	May be present	Absent	Absent
Calcification	May occur	Occasional, nonspecific	Rare	Rare
Intraconal Extension	Rare	Possible in advanced disease	–	–

## 11 Conclusion

Lacrimal gland masses encompass a diverse and diagnostically challenging group of pathologies, ranging from benign and malignant neoplasms to inflammatory, autoimmune, and systemic disorders. Radiological imaging, particularly MRI and CT, plays a vital role in the localisation, characterisation, and differentiation of these lesions. A detailed understanding of the key imaging features, including lesion composition, lobe predilection, enhancement patterns, and associated bony involvement, can significantly improve preoperative diagnostic accuracy, and guide appropriate clinical decision-making.
